# Synaptic loss in schizophrenia: a meta-analysis and systematic review of synaptic protein and mRNA measures

**DOI:** 10.1038/s41380-018-0041-5

**Published:** 2018-03-06

**Authors:** Emanuele Felice Osimo, Katherine Beck, Tiago Reis Marques, Oliver D Howes

**Affiliations:** 1 Psychiatric Imaging Group, MRC London Institute of Medical Sciences, Hammersmith Hospital, Imperial College London, London, UK; 2 Psychiatric Imaging Group, Institute of Clinical Sciences, Faculty of Medicine, Imperial College London, London, UK; 3 Department of Psychiatry, University of Cambridge, Cambridge, UK; 4 Cambridgeshire and Peterborough NHS Foundation Trust, Cambridge, UK; 5 Department of Psychosis Studies, Institute of Psychiatry, Psychology and Neuroscience, King’s College London, London, UK; 6 South London and Maudsley NHS Foundation Trust, Camberwell, London, UK

**Keywords:** Schizophrenia, Biological techniques

## Abstract

Although synaptic loss is thought to be core to the pathophysiology of schizophrenia, the nature, consistency and magnitude of synaptic protein and mRNA changes has not been systematically appraised. Our objective was thus to systematically review and meta-analyse findings. The entire PubMed database was searched for studies from inception date to the 1st of July 2017. We selected case-control postmortem studies in schizophrenia quantifying synaptic protein or mRNA levels in brain tissue. The difference in protein and mRNA levels between cases and controls was extracted and meta-analysis conducted. Among the results, we found a significant reduction in synaptophysin in schizophrenia in the hippocampus (effect size: −0.65, *p* < 0.01), frontal (effect size: −0.36, *p* = 0.04), and cingulate cortices (effect size: −0.54, *p* = 0.02), but no significant changes for synaptophysin in occipital and temporal cortices, and no changes for SNAP-25, PSD-95, VAMP, and syntaxin in frontal cortex. There were insufficient studies for meta-analysis of complexins, synapsins, rab3A and synaptotagmin and mRNA measures. Findings are summarised for these, which generally show reductions in SNAP-25, PSD-95, synapsin and rab3A protein levels in the hippocampus but inconsistency in other regions. Our findings of moderate–large reductions in synaptophysin in hippocampus and frontal cortical regions, and a tendency for reductions in other pre- and postsynaptic proteins in the hippocampus are consistent with models that implicate synaptic loss in schizophrenia. However, they also identify potential differences between regions and proteins, suggesting synaptic loss is not uniform in nature or extent.

## Introduction

Schizophrenia is a chronic mental illness, affecting ~1% of the population [1, 2]. Imaging studies have demonstrated that schizophrenia is associated with ventricular enlargement [3, 4], a whole brain volume reduction of around 3%, and regional volume reductions of 6–10% in grey matter areas such as the frontal cortex [5, 6] and hippocampus [7–10], as well as alterations in astroglial markers [11, 12]. However, histopathological work has failed to find clear evidence of gliosis or other degenerative changes in schizophrenia, and, while there is cortical volume loss, this occurs in the absence of neuronal cell loss [13–18]. Instead, it has been suggested that lower grey matter volumes are due to a reduction in synaptic levels, which would be compatible with the neurodevelopmental hypothesis of schizophrenia [2, 19–22].

**Fig. 1:**
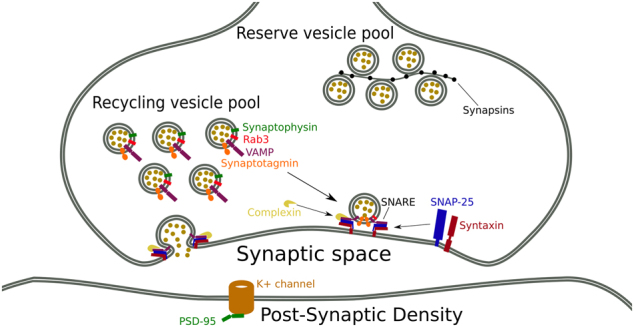
Showing the location of synaptic proteins in the synapses. Rab3 Ras-related protein, VAMP vesicle-associated membrane protein, also known as synaptobrevin, SNAP-25 synaptosomal-associated protein 25, PSD-95 postsynaptic density protein 95, SNARE SNap REceptor complex

A number of proteins expressed in presynaptic terminals and the postsynaptic density (Fig. 1) are used as markers of synaptic density [23–26]. Synaptophysin is the most studied presynaptic protein, and an accurate index of neuronal synaptic density [27] because it is limited to neuronal synapses [24]. This protein interacts with synaptobrevin, thus participating in synaptic vesicle exocytosis [28]. It is specifically enriched in presynaptic nerve terminals, and is integral to the synaptic vesicle membrane [29, 30]. Consequently, it has been widely used in the quantification of synapses in human postmortem cortical samples [24, 25]. Other synaptic markers include the SNap Receptor (SNARE) complex proteins, comprising SNAP-25 (Synaptosomal-associated protein 25), syntaxin and vesicle-associated membrane protein (VAMP), also known as Synaptobrevin. The SNARE complex is crucial for calcium-dependent exocytosis at chemical synapses and is required for dopaminergic, serotonergic [31] and glutamatergic function [32]. Given the potential role of these systems in schizophrenia [33, 34], this makes the SNARE complex of particular interest. Synaptophysin and SNARE complex proteins are depleted in conditions associated with synaptic loss, such as Alzheimer’s disease, other dementias and epilepsy [35–37]. Complexins are presynaptic membrane proteins that bind syntaxin, and are thought to be SNARE modulators. Complexin I is enriched in inhibitory neurons, while Complexin II is more commonly found in excitatory neurons [38, 39]. Synapsin I and II are proteins involved in neurite elongation and synapse formation and maintenance [40]; synapsin III is also a modulator of plasticity processes and of dopaminergic function [41]. Rab3A (Ras-related protein Rab-3A) and synaptotagmin are both involved in regulating synaptic vesicle exocytosis [42, 43]. PSD-95 (postsynaptic density protein 95) is abundant in the brain and concentrated in the postsynaptic density (PSD). It has been implicated in forming and maintaining excitatory synapses [44, 45], and in regulating synaptic strength and plasticity by interacting with other synaptic proteins, including glutamate receptors [46].

To our knowledge, there has not been a previous meta-analysis of synaptic protein levels in schizophrenia. We therefore aimed to synthesise the postmortem findings in patients with schizophrenia and healthy controls, and then discuss the implications of these findings in relation to the pathophysiology of the disorder.

## Methods and materials

### Data extraction

The main outcome measure was the difference in synaptic protein and mRNA levels between patients with schizophrenia and healthy controls. In addition, we extracted the following variables: sample size, methods of quantification, inclusion criteria, mean age, patients’ medication, postmortem interval (PMI), cause of death, percentage of suicides, and whether the analysis was blind to group status.

### Statistical analysis

We performed a meta-analysis when there were at least 5 independent data sets in each specific brain region, as recommended for meta-analyses using random-effects approaches [47].

The main outcome measure was the effect size (ES) (Hedges’ *g*) of synaptic protein/mRNA change in patients with schizophrenia and healthy controls for each reported region or sub-region of interest. See Supplementary Information for further methodological details.

## Results

The literature search yielded 281 results, from which we identified 60 relevant papers (see Supplementary Figure 1 for the PRISMA diagram of the literature search). 36 of the 60 studies met criteria for inclusion in the quantitative synthesis. We were able to perform a meta-analysis of synaptophysin protein levels for hippocampus, frontal cortex, cingulate cortex (CC), temporal cortex and occipital cortex. In the frontal cortex, it was possible to perform a meta-analysis of the following synaptic proteins: synaptophysin, SNAP-25, PSD-95, VAMP, and syntaxin. All studies included in the meta-analyses-matched cases and controls for age at death except for one [48], and postmortem interval (PMI) was matched in 31 out of 36 studies. 19 out of the 36 studies (52.8%) reported that the experimenter was blind to diagnosis while conducting their analyses. See supplementary Tables 1–9 for these and further details of the studies [48–107].

There were insufficient data for meta-analysis of mRNA data in any brain region. Instead the results from the individual studies of mRNA and protein levels, where there were insufficient studies for meta-analysis are summarised below and in Supplementary Tables 1–9.

### Synaptophysin levels in the hippocampus

**Fig. 2:**
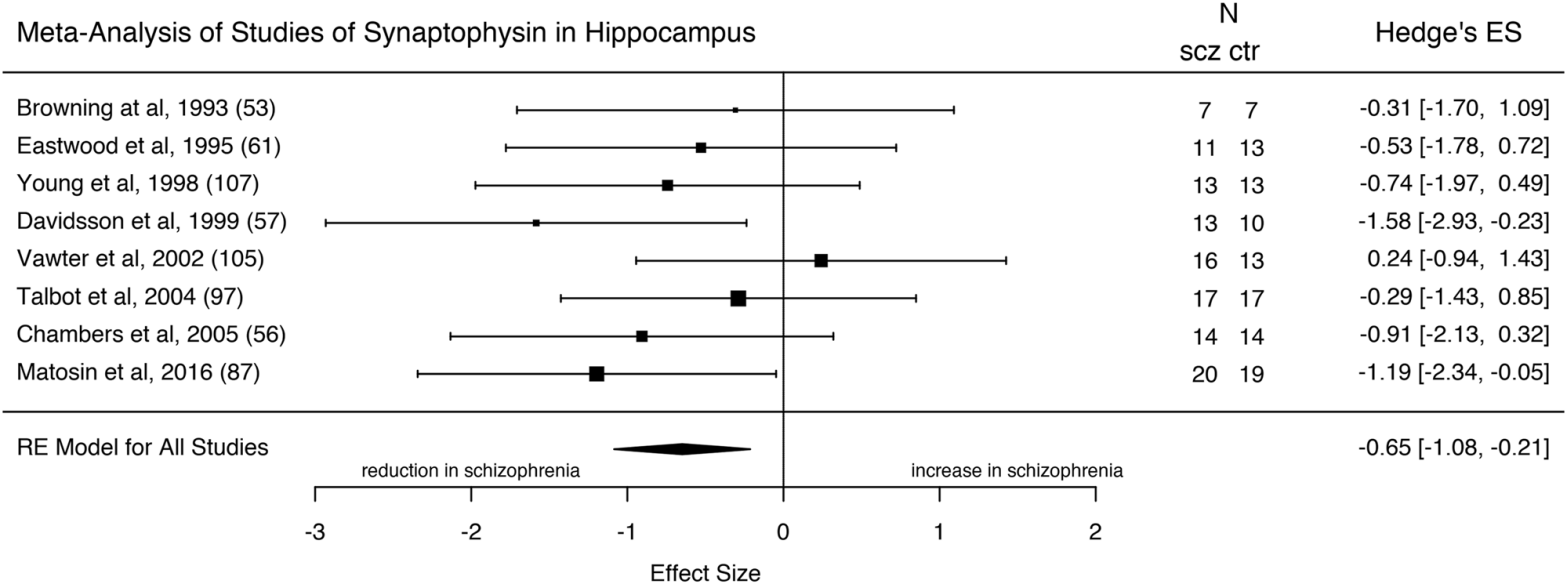
Forest plot showing the effect sizes for studies of synaptophysin in hippocampus in schizophrenia patients as compared to controls. There was a significant reduction in schizophrenia (effect size = −0.65, *p* = 0.0036)

Eight studies (111 patients with schizophrenia and 106 healthy controls) measured synaptophysin levels in the hippocampus (CA1–4 and Dentate Gyrus). Synaptophysin was significantly reduced in patients with schizophrenia with an ES of −0.65 (Fig. 2; *z* = −2.91; 95% confidence interval (CI) = −1.08, −0.21; *p* = 0.0036). The *I*^2^ statistic revealed low heterogeneity (*I*^2^ = 0%; 95% CI = 0, 70.5; Cochrane’s *Q* = 5.7; *p* = 0.57). The funnel plot appeared symmetrical, and a regression test for funnel plot asymmetry was non-significant (*z* = −0.54; *p* = 0.59), suggesting there is no evidence of publication bias (Supplementary Figure 2). The studies were well matched for PMI and the meta-regression for the proportion of suicides was not significant (*p* = 0.83, for the studies where suicide data were available), suggesting this was not a major bias. Of the two mRNA studies of synaptophysin in the hippocampus, one showed significantly decreased synaptophysin mRNA levels in schizophrenia, the other a non-significant reduction [59, 106] (see Supplementary Table 1).

### Summary of findings with other synaptic proteins and mRNAs

In the hippocampus, three studies examined SNAP-25 protein levels, two of which found a significant reduction in schizophrenia. Three studies measured PSD-95 protein levels, one of which found a significant reduction, the other found a trend reduction in schizophrenia. For the complexins, two studies measured protein levels and found no change, and two studies measured mRNA levels separately for complexin I (which was only reduced in some subfields) and complexin II (which was significantly reduced overall in one study, and in some subfields in the other). Four studies measured synapsin protein levels, three of which found a significant reduction in schizophrenia. Rab3A protein levels were studied twice and both times were found significantly reduced in schizophrenia.

### Synaptic proteins and mRNA levels in frontal cortex

#### Synaptophysin

**Fig. 3:**
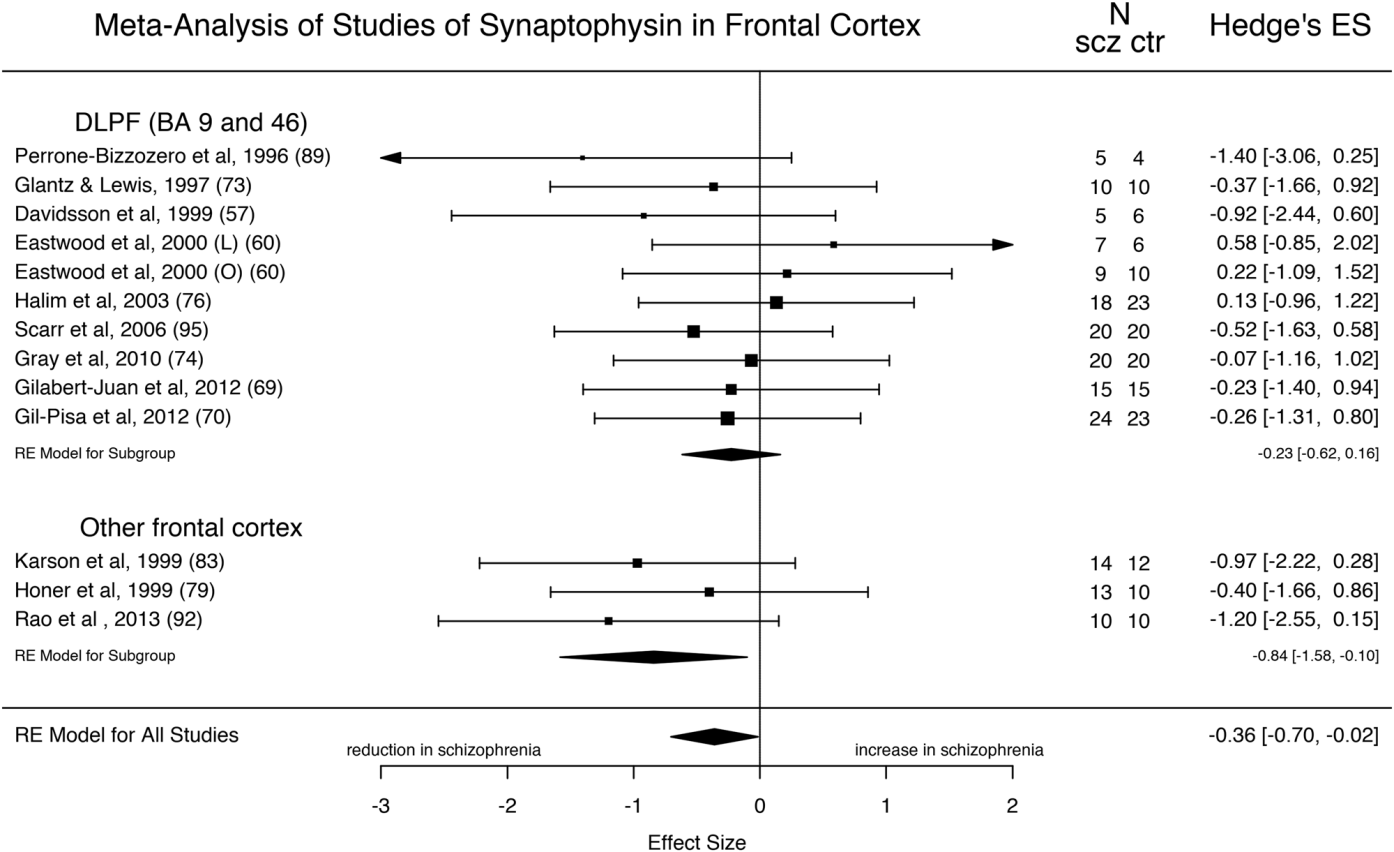
Forest plot showing the effect sizes for studies of synaptophysin in frontal cortex in schizophrenia patients as compared to controls. There was a significant reduction in schizophrenia (effect size = −0.36, *p* = 0.04)

Thirteen studies comprising 170 patients with schizophrenia and 169 healthy controls measured synaptophysin levels in frontal cortical regions (approximating Brodmann Areas 9, 10, 46, 47) (Fig. 3). The majority of studies of the frontal cortex examined the dorso-lateral pre-frontal cortex (DLPFC; approximating BAs 9 and 46) [57, 60, 69, 70, 73, 74, 76, 89, 95], while three studies examined BAs 10 and 45 [79, 83, 92]. Synaptophysin was significantly reduced in patients with schizophrenia with an ES of −0.36 (*z* = −2.05; 95% CI = −0.70, −0.02; *p* = 0.04). The *I*^2^ statistic revealed low heterogeneity (*I*^2^ = 0%; 95% CI = 0–50.1%; Cochrane’s *Q* = 8.1; *p* = 0.78). Inspection of the funnel plot did not reveal asymmetry (Supplementary Figure 3), and the regression test for funnel plot asymmetry was non-significant (*z* = −1.15; *p* = 0.25). A sub-analysis only including studies relating to the DLPFC was non-significant (ES = −0.23; *z* = −1.14; *p* = 0.25), while the number of studies investigating other frontal areas was not sufficient for a separate sub-analysis. An exploratory meta-regression of the effect of the percentage of suicides on the ES for the studies where this information was available showed no significant effect (*p* = 0.98). PMI was significantly different between cases and controls in one study [69]. In case this was biasing the results, we excluded this study and re-ran the meta-analysis, finding the reduction in synaptophysin levels remained significant (ES = −0.37; *z* = −2.03; *p* = 0.04). With regards to mRNA data for synaptophysin in the frontal cortex, one study reports a significant reduction in schizophrenia, while one reports a significant reduction in BAs 17 and 22 and a non-significant reduction in BAs 9 and 46, and two studies suggest no change in frontal cortex (Supplementary Table 1).

### SNAP-25

**Fig. 4:**
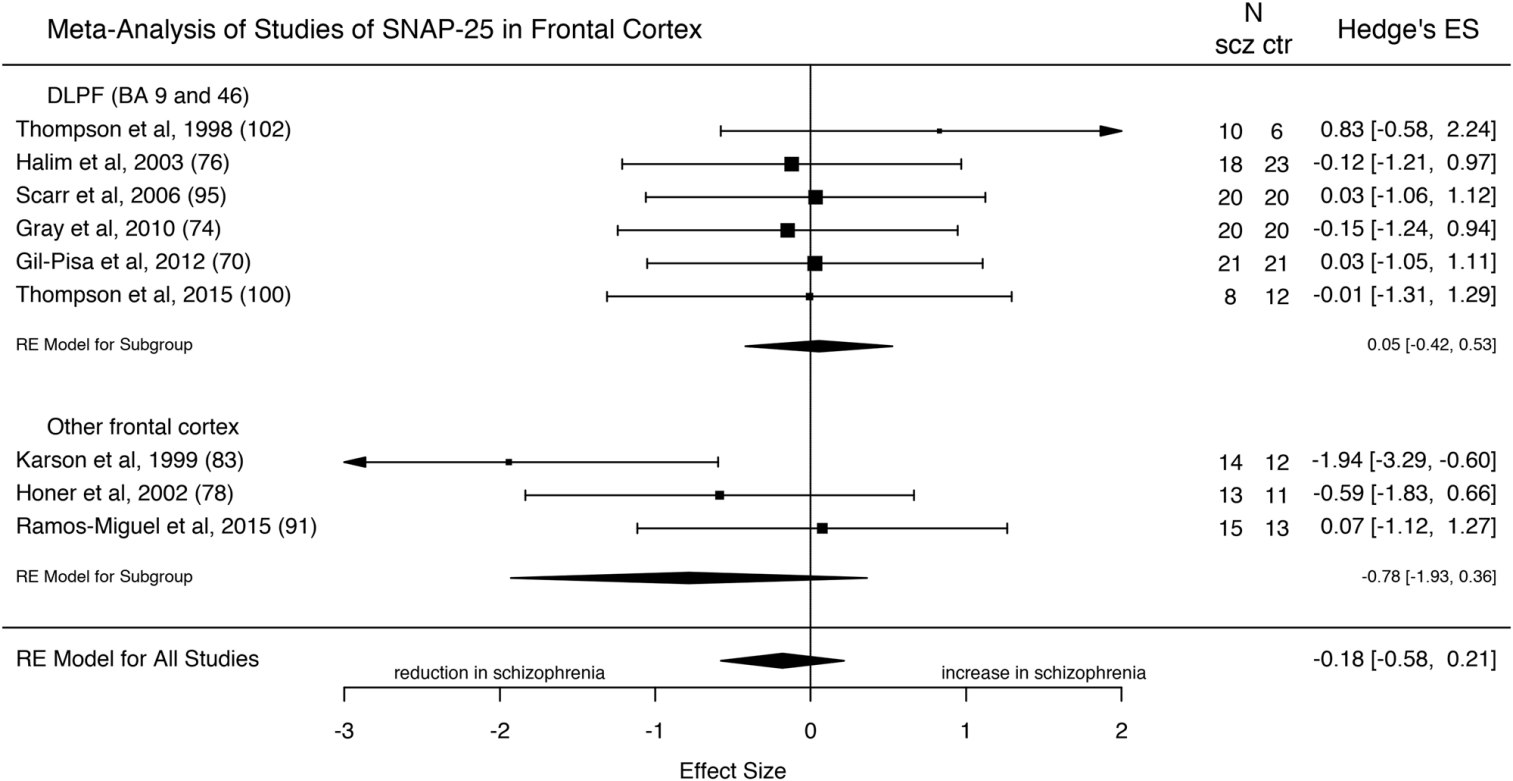
Forest plot showing the effect sizes for studies of SNAP-25 in frontal cortex in schizophrenia patients as compared to controls. There was no significant reduction in schizophrenia (effect size = −0.18, *p* = 0.37)

Nine studies comprising 139 patients with schizophrenia and 138 controls measured SNAP-25 levels in frontal cortex (approximating BAs 9, 10, 46, 47) (Fig. 4). The overall results indicate no significant change in SNAP-25 in frontal cortex in schizophrenia (ES: −0.18; *z* = −0.90; 95% CI = −0.58, 0.21; *p* = 0.37). The *I*^2^ statistic revealed low heterogeneity (*I*^2^ = 0%; 95% CI = 0–81%; Cochrane’s *Q* = 9.5; *p* = 0.30). The three mRNA studies of SNAP-25 in the frontal cortex showed non-significant reductions or no changes in mRNA levels in schizophrenia (Supplementary Table 2).

### PSD-95, VAMP, and syntaxin

PSD-95 (6 studies, ES = −0.34, *p* = 0.14), VAMP (6 studies, ES = −0.26, *p* = 0.27), and syntaxin (6 studies, ES = 0.16, *p* = 0.52) protein levels did not differ in frontal cortex between schizophrenia patients and controls (Supplementary Figures 4–6). Of the four mRNA studies of PSD-95 in the frontal cortex, two showed no change, one a non-significant reduction and one non-significant increase in mRNA levels in schizophrenia (Supplementary Table 3). One study measured VAMP mRNA levels and found no difference in frontal cortex (Supplementary Table 5). Our search did not identify any studies of syntaxin mRNA in frontal cortex.

### Summary of findings with other synaptic proteins and mRNAs

For frontal cortex, four studies measured levels of the complexins: one of the two studies looking at protein levels found a significant reduction in complexin I in schizophrenia, and one of the studies looking at mRNA levels found a reduction in complexin II. Three studies measured synapsin protein levels, and one found a significant reduction in synapsin III, while of the two studies quantifying mRNA, one found a significant reduction in synapsin II in schizophrenia. Both studies of Rab3A found a significant reduction in protein levels in frontal cortex. No change was found in protein levels in schizophrenia in two studies for synaptotagmin in this region.

### Synaptophysin levels in cingulate cortex

**Fig. 5:**
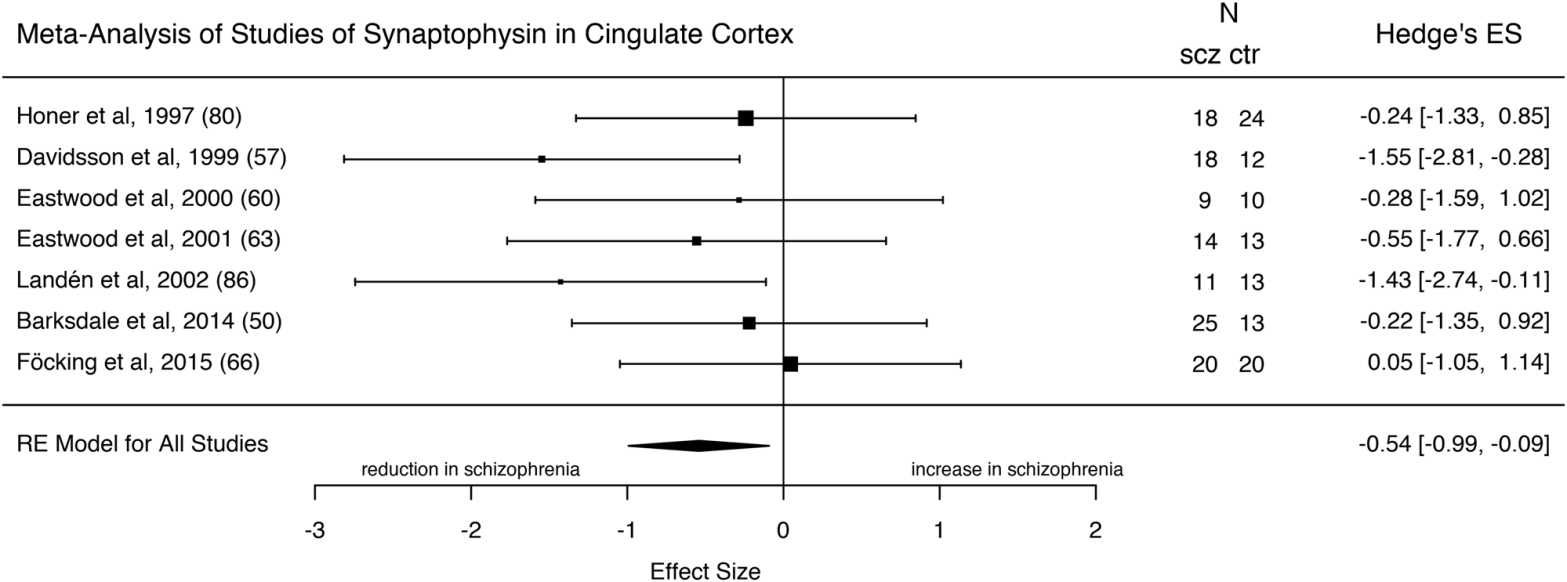
Forest plot showing the effect sizes for synaptophysin levels in the cingulate cortex in schizophrenia patients as compared to controls. There was a significant reduction in schizophrenia (effect size = −0.54, *p* = 0.02)

Seven studies (comprising 115 patients with schizophrenia and 105 healthy controls) measured synaptophysin in the CC (approximating BAs 24, 32, 33). Synaptophysin was significantly reduced in the CC of patients with schizophrenia with an ES of −0.54 (Fig. 5; *z* = −2.35; 95% CI = −0.99, −0.09; *p* = 0.02). The *I*^2^ statistic revealed low heterogeneity (*I*^2^ = 0%; CI = 0, 80.4; Cochrane’s *Q* = 6.0; *p* = 0.42). Inspection of the funnel plot suggested a degree of asymmetry, however, the regression test for funnel plot asymmetry was non-significant (*z* = −1.78; *p* = 0.07) (Supplementary Figure 7), and the trim and fill analysis did not reveal any missing studies. There was insufficient information to test the effect of suicide as a meta-regressor. While the majority of studies reported samples coming from the anterior CC (ACC), Honer et al. [80] describe their sample as from the CC without specifying a particular sub-region. A sub-analysis removing this study shows that there still is a significant reduction in synaptophysin levels in the ACC in schizophrenia relative to controls (ES = −0.61; *z* = −2.27; 95% CI = −1.14, −0.08; *p* = 0.02). In further sensitivity analyses, removing the study by Landén et al. [86], which shows a significant difference in PMI between cases and controls, affects the overall significance (ES = −0.42; *z* = −1.73; CI = −0.90, 0.06; *p* = 0.08). Our search did not find a study of synaptophysin mRNA in this region.

### Summary of findings with other synaptic proteins and mRNAs in cingulate cortex

Two studies measured SNAP-25 protein levels in the CC, and found no significant change. With regards to PSD-95, three studies measured protein levels in CC: two found a significant reduction, the other no change in schizophrenia, while one study found a significant increase in PSD-95 mRNA in this area. One study measured the complexins in this area and found no change. Both studies of Rab3A found a significant reduction in protein levels in CC in schizophrenia.

### Synaptophysin levels in temporal cortex

Six series in five studies (60 patients and 57 controls) measured synaptophysin protein levels in the temporal cortex. There were no significant differences in synaptophysin levels in schizophrenia patients when compared to healthy controls in the temporal cortex (ES = −0.31; *z* = −1.12; 95% CI = −0.85, 0.23; *p* = 0.26—Supplementary Figure 8). Synaptophysin mRNA were found significantly decreased in two of three studies of this molecule in temporal cortex.

### Summary of findings with other synaptic proteins and mRNA levels in temporal cortex

SNAP-25 protein levels were found significantly decreased in one of two studies, while mRNA levels were unchanged in one study in schizophrenia. Syntaxin, VAMP, synapsin, Rab3A, and synaptotagmin mRNA levels were not significantly altered in one study each. Rab3A protein levels were unchanged in two studies in temporal cortex. For the complexins, one study analysed protein levels and found a reduction in complexin II only. For complexin mRNAs, three studies reported reductions in complexin II, while no study found significant reductions in complexin I.

### Synaptophysin and other protein and mRNA levels in occipital cortex

Five series in four studies (51 patients and 48 controls) measured synaptophysin protein levels in the occipital cortex. There were no significant differences in synaptophysin levels in schizophrenia patients when compared to healthy controls in the occipital cortex (ES = −0.16; *z* = −0.45; 95% CI = −0.84, 0.52; *p* = 0.65—Supplementary Figure 9). One study measured synaptophysin mRNA levels in the occipital cortex, and found a significant reduction. Two studies measured PSD-95 in occipital cortex in schizophrenia: one found a significant increase in its mRNA, and one found no change in PSD-95 protein levels. There were insufficient studies for meta-analysis of other synaptic protein or mRNA levels in this region.

## Discussion

Our main findings are that protein levels of the synaptic marker synaptophysin are significantly decreased in schizophrenia in the hippocampus and cingulate cortex. We also found a decreased level of synaptophysin mRNA levels in the hippocampus [59, 106] (see Supplementary Table 1).

The frontal cortex also shows a significant reduction in synaptophysin protein levels. However, the ES is numerically smaller than for hippocampus and CC. Moreover, the sub-analysis restricted to the DLPFC was not significant, and the mRNA data for synaptophysin in the frontal cortex are inconsistent, with two studies suggesting a reduction, and two studies suggesting no change (Supplementary Table 1). Furthermore, the other protein levels in frontal cortex that we meta-analysed (SNAP-25, PSD-95, VAMP, and syntaxin) are not significantly reduced. Taken together, this suggests findings are less consistent in the frontal cortex than the findings in the hippocampus and CC. Among the potential contributors to these inconsistencies are age [108] and mode of death [109, 110]. However, all of our studies matched the subjects for age at death, and our meta-regressions for suicide as manner of death were all not significant, suggesting this is unlikely to be a major contributor to inconsistency. Other potential explanations for these inconsistencies could be differences in protein quantification methodology, variations in dissection protocols, and differences in the biological substrate due to the heterogeneity of the illness being studied, in addition to sub-regional variability (as suggested by lack of difference in the DLPFC); we discuss each of these sources of variation in the methodological section below; see also the review by McCullumsmith and colleagues for a further discussion of the factors that may influence postmortem findings [109]. We found no evidence of synaptic protein change in the temporal and occipital cortex. Our meta-analytic findings are summarised in Table 1.

**Table 1:** Summary of our meta-analytic results

Protein: area:	synaptophysin	SNAP-25	PSD-95	VAMP	syntaxin
Hippocampus	↓ −0.65	N/A	N/A	N/A	N/A
Cingulate cortex	↓ −0.54	N/A	N/A	N/A	N/A
Frontal cortex	↓ −0.36	↔ −0.18	↔ −0.34	↔ −0.26	↔ 0.16
Temporal cortex	↔ −0.31	N/A	N/A	N/A	N/A
Occipital cortex	↔ −0.16	N/A	N/A	N/A	N/A

The number is the effect size (Hedges’ *g*) and “↓” indicates a significant reduction in schizophrenia, while “↔“ indicates no significant difference

*N/A* not available

### Interpretation of findings

Our findings of reductions in synaptophysin levels extend postmortem microscopy studies in schizophrenia that have found synaptic loss in the hippocampus [111, 112] and ACC [113] by providing meta-analytic evidence consistent with loss of synapses between neurons. They also extend a meta-analysis of genetic expression studies that found that genes in the presynaptic secretory function category (including synaptophysin) were significantly altered in schizophrenia [114], by providing evidence that this translates into alterations in protein levels of synaptophysin. Interestingly, the brain areas we found to have lower synaptophysin levels are among the regions that show the most volume loss in schizophrenia [115–120]. There is evidence that this cortical loss is at least partially due to reduced neuropil, including reduced synaptic compartments, rather than neuronal loss [121]. It is therefore possible that the reductions in the synaptic marker observed in our meta-analysis indicate that loss of synapses contributes to the brain volume loss seen in imaging and postmortem studies. Consistent with this, volume loss in hippocampus in schizophrenia is present from the onset of symptoms, pre-dates antipsychotic exposure, and does not appear to be secondary to neuron loss [7, 18, 122–124], occurring in the absence of a change in total neuron numbers [15, 18]. However, it should be recognised that there is considerable debate about the cellular changes that underlie brain volume alterations in schizophrenia, and other cellular changes, including alterations in axonal density, glial cells and neuronal size could also contribute to loss of neuropil [121]. The role of synaptic alterations and contribution of these other factors to volume loss needs further testing. Postmortem studies of the CC in schizophrenia have also found structural alterations, including synaptic loss [113, 125].

We found no significant changes in synaptic density in some of the brain areas studied in this meta-analysis, such as temporal and occipital cortices. Taken with our findings of significant reductions in hippocampus, cingulate and frontal cortex, this could suggest that synaptic loss shows regional specificity, affecting some areas more than others, which is similar to the pattern of regional volume changes in schizophrenia [10, 120]. This is consistent with models of schizophrenia that implicate the hippocampus and frontal cortex as central to the pathophysiology of the disorder [5, 7, 118, 123, 125–130]. However, while the lack of significant differences in the temporal and occipital cortex raises the question of what underlies the grey matter volume reductions commonly reported in these regions [120, 131], we caution about over-interpretation of regional differences as there is a risk of a type II error. Recent work has also suggested a temporal specificity of synaptic change in schizophrenia, with synaptogenesis predominating earlier in the disease, and synaptic loss in chronic phases [132]. Ultimately, further studies are needed to compare sub-regions and timing with regards to disease onset.

Although there is some evidence that synaptophysin might be more abundant in glutamatergic than in GABAergic vesicles [133], it should be noted that it is not specific enough to particular synapses to draw firm conclusions. Thus, the reductions may reflect a global loss of synapses or be specific to particular neuronal populations.

Our findings of a significant reduction in frontal cortex in synaptophysin but not other synaptic markers is intriguing. Synaptophysin is specific to presynaptic nerve terminals [29, 30]. It binds cholesterol, which is required for the genesis of synaptic vesicles [134]. This could indicate dysfunction in vesicle formation. Synaptophysin is considered one of the best proxies for synaptic density [27], and may be more sensitive to detecting synaptic reductions than the other markers, so the lack of reductions in the other markers could be a type II error. Ultimately, large studies comparing multiple synaptic marker levels across brain regions are required to definitively test whether there is greater reduction in some regions, such as the hippocampus, and proteins relative to other regions and proteins.

### Methodological considerations

A potential limitation of this meta-analysis is that studies used different methods of protein quantification (24 studies used western blotting (WB), 7 immunohistochemistry and 5 using ELISA—see Supplementary Information). However, a study comparing the different techniques for assessing synaptophysin levels in brain tissue found that WB and immunohistochemistry methods give similar results [27]. Another study compared WB and ELISA for synaptophysin quantification found that ELISA might be more robust at synaptophysin quantitation [135]. However, combining different methods with different levels of precision and sensitivity in the same meta-analysis should not account for our findings of reductions in schizophrenia, as the degree of precision is taken into account by the measure of dispersion, and variability in this would reduce the sensitivity to detect differences between groups, if it had any effect. Furthermore, we have used a random-effects model approach, which takes into account inter-study variability. However, we cannot exclude that our findings of no significant differences in the other regions examined could be a type II error due to variability in the sensitivity of methods used, and the smaller number of studies that assessed these areas, meaning that our meta-analysis may have been under-powered to detect small effects. Further studies are needed in these regions to rule this possibility out.

A potential confounder in the studies included is the use of antipsychotic medication in samples. There is evidence to suggest that antipsychotics may cause brain structural abnormalities, such as striatal [136] or brain volumetric changes [137]. However, studies have shown no difference in synaptophysin levels in the hippocampus of rats after antipsychotic exposure [59]; it should be noted that the animals used in these experiments were healthy animals, and could therefore not fully reflect results in schizophrenia. Similar studies on the frontal cortex and striatum have shown either no change or an increase in synaptophysin following antipsychotic treatment [138–140]. In addition, non-human primate studies have shown that synaptophysin levels are not affected following the continuous administration of haloperidol for several weeks [141, 142]. Thus, we find that antipsychotic treatment is unlikely to account for the reductions in synaptophysin, but studies in antipsychotic-naïve patients are required to definitively rule an effect out. Studying lifetime antipsychotic dose as a meta-regressor was not possible in the present study as this information was not present in the majority of studies. Unfortunately, it was also impossible to study illness duration as a meta-regressor as this information was not present in the majority of the included studies.

PMI was significantly different between groups in 5 out of 36 studies. When the one non-matched PMI study was removed from the analysis of synaptophysin in the frontal cortex, it did not affect the overall significance. However, in the analysis of synaptophysin in CC, after removing the study that did not match groups for PMI [86], the overall effect was no longer significant, suggesting that differences in PMI may contribute to differences in this region.

We were able to explore the potential effect of suicide on our findings because it was widely reported, but this was not possible for other potential contributors to inconsistency because they were not consistently reported. This should not be taken as indicating they are not important, and it is recommended that future studies report these in more detail to facilitate comparisons.

Other potential sources of variability are the differences in laterality [143, 144], dissection protocols and tissue processing. However, few studies reported data by hemisphere, precluding analysis of potential differences. Tissues sources are summarised in the Supplementary Tables; unfortunately, few papers mention the dissection protocol that was used, therefore it was impossible for us to take this factor into account. In addition to this, the brains came from different sources: some samples came from brain banks, which collect samples from different consortia, each with different dissection protocols; some papers sourced their own samples without specifying the dissection technique they used, and for 17 samples the source was not mentioned.

There is evidence that there may be variability in gene expression depending on the specific dissection boundaries [145]. Some of the studies we included used immunohistochemistry to quantify synaptic proteins, and reported protein and mRNA levels for different tissue layers and/or very specific sub-regions within the same region, thus, also confirming that molecular profiles within brain regions vary on a gradient [39, 56, 64, 65, 69, 73, 93, 97, 103]. Other studies used tissue homogenates, therefore, in our meta-analysis, we combined the data from different sub-regions within a given region, which could obscure sub-regional differences, as suggested by analyses of grey matter alterations [144].

### Future directions

Our findings raise a number of questions. In particular, whether the reduction in synaptophysin is developmental or develops later in life; whether it is primary or secondary to other factors and changes, such as oxidative stress [146] or inflammation [147, 148]; whether it indicates a loss of synapses or the loss of synaptophysin specifically, and how it relates to grey matter changes and symptoms. Further studies are needed to tackle these questions. The recent development of PET tracers that index synaptic proteins provides a means of addressing some of them. Longitudinal in-vivo imaging studies with synaptic tracers, from childhood to early in the course of illness to a chronic stage, are needed to address the questions relating to the time course of the changes. The concomitant study of other biological factors of the illness, such as oxidative stress, inflammation and structural brain changes, would allow the correlations of these elements with synaptic loss to be tested. This work would need to be complemented by preclinical studies to determine the effect of these potential mechanisms on synaptic proteins that can be measured postmortem and in vivo using PET imaging.

Finally, these results may also have implications for drug development. In animal models, the administration of a p21-activated kinases (PAK) inhibitor in late adolescence has been shown to block synaptic loss and prevent adult behavioural deficits associated with schizophrenia [149]. Reversing or preventing synaptic loss could therefore be a potential treatment target in schizophrenia.

## Conclusions

There is a significant reduction in synaptophysin in the hippocampus, cingulate and frontal cortices of patients with schizophrenia as compared to matched healthy controls, although the findings in the CC were not significant after excluding a study that did not match for PMI, and we did not find significant results for the levels of SNAP-25, PSD-95, VAMP and syntaxin in the frontal cortex. We found no difference in temporal cortex and occipital cortex for synaptophysin. These findings are consistent with models that implicate synaptic loss in hippocampus and frontal cortical regions in the pathophysiology of schizophrenia, but further studies are required to determine if this is a general loss of synapses or specific loss of synaptophysin, and to test regional variability.

## Electronic supplementary material

Supplementary Information
