# Supplementary material for: Synaptic loss in schizophrenia: a meta-analysis and systematic review of synaptic protein and mRNA measures
Source: Mol Psychiatry. 2018 Mar 6;24(4):549–61. doi: 10.1038/s41380-018-0041-5 (PMC6004314; doi:10.1038/s41380-018-0041-5)
Supplement: Supplementary file 1 — Supplementary Information [file 41380_2018_41_MOESM1_ESM.docx]

SUPPLEMENTARY INFORMATION

Table of Contents

[Supplementary Methods: 3](#_Toc497292077)

[SEARCH STRATEGY, STUDY SELECTION, INCLUSION AND EXCLUSION CRITERIA: 3](#_Toc497292078)

[DATA EXTRACTION: 3](#_Toc497292079)

[STATISTICAL ANALYSIS: 3](#_Toc497292080)

[Supplementary Tables 5](#_Toc497292081)

[Supplementary Table 1: Summary of the studies of synaptophysin levels in schizophrenia: sample and methodological characteristics and findings 5](#_Toc497292082)

[Supplementary Table 2: Summary of the studies of SNAP-25 levels in schizophrenia: sample and methodological characteristics and findings 8](#_Toc497292083)

[Supplementary Table 3: Summary of the studies of PSD-95 levels in schizophrenia: sample and methodological characteristics and findings 10](#_Toc497292084)

[Supplementary Table 4: Summary of the studies of syntaxin levels in schizophrenia: sample and methodological characteristics and findings 11](#_Toc497292085)

[Supplementary Table 5: Summary of the studies of VAMP (synaptobrevin) levels in schizophrenia: sample and methodological characteristics and findings 11](#_Toc497292086)

[Supplementary Table 6: Summary of the studies of complexin levels in schizophrenia: sample and methodological characteristics and findings 12](#_Toc497292087)

[Supplementary Table 7: Summary of the studies of synapsins levels in schizophrenia: sample and methodological characteristics and findings 13](#_Toc497292088)

[Supplementary Table 8: Summary of the studies of Rab3A levels in schizophrenia: sample and methodological characteristics and findings 14](#_Toc497292089)

[Supplementary Table 9: Summary of the studies of synaptotagmin levels in schizophrenia: sample and methodological characteristics and findings 15](#_Toc497292090)

[Supplementary Figures 16](#_Toc497292091)

[**Supplementary Figure 1:** Flowchart showing the inclusion of studies for meta-analysis. 16](#_Toc497292092)

[**Supplementary figure 2:** Meta-analysis funnel plot of synaptophysin in hippocampus in schizophrenia patients as compared to controls. 17](#_Toc497292093)

[**Supplementary figure 3:** Meta-analysis funnel plot of synaptophysin in frontal cortex in schizophrenia patients as compared to controls. 17](#_Toc497292094)

[**Supplementary Figure 4:** Forest plot showing the effect sizes for studies of PSD-95 in frontal cortex in schizophrenia patients as compared to controls. There was no significant reduction in schizophrenia (effect size= -0.34, p= 0.14). 18](#_Toc497292095)

[**Supplementary Figure 5:** Forest plot showing the effect sizes for studies of VAMP in frontal cortex in schizophrenia patients as compared to controls. There was no significant reduction in schizophrenia (effect size= -0.26, p= 0.27). 18](#_Toc497292096)

[**Supplementary Figure 6:** Forest plot showing the effect sizes for studies of syntaxin in frontal cortex in schizophrenia patients as compared to controls. There was no significant reduction in schizophrenia (effect size= 0.16, p= 0.52). 18](#_Toc497292097)

[**Supplementary figure 7:** Meta-analysis funnel plot if synaptophysin in cingulate cortex in schizophrenia patients as compared to controls. 19](#_Toc497292098)

[**Supplementary Figure 8:** Forest plot showing the effect sizes for synaptophysin levels in the temporal cortex in schizophrenia patients as compared to controls. There was no significant reduction in schizophrenia (effect size= -0.31, p= 0.26) 19](#_Toc497292099)

[**Supplementary Figure 9:** Forest plot showing the effect sizes for synaptophysin levels in the occipital cortex in schizophrenia patients as compared to controls. There was no significant reduction in schizophrenia (effect size= -0.16, p= 0.65) 20](#_Toc497292100)

[SUPPLEMENTARY REFERENCES: 21](#_Toc497292101)

## Supplementary Methods:

### SEARCH STRATEGY, STUDY SELECTION, INCLUSION AND EXCLUSION CRITERIA:

We searched PubMed for papers published from onset of the databases until the 1^st^ of July 2017. The reference sections of all retrieved articles were manually searched for additional studies. To be included in this meta-analysis, an article needed to investigate one of the widely used and well validated synaptic markers (synaptophysin, SNAP-25, syntaxin, VAMP, complexins, synapsin, rab3a, synaptotagmin, or PSD-95) or their corresponding mRNAs in the postmortem brains of patients with schizophrenia as compared to matched healthy controls.

We used an *a priori* search strategy, with the aim of including all publications relevant to our subject. Reference lists and abstracts of the papers identified by the search were used to identify further publications meeting the selection criteria.

The abstracts of the studies identified by the search were screened by one researcher (EFO) and the screening was independently repeated by a second author (KB) to check all potential articles were included. Inclusion criteria were: 1) original articles reporting human postmortem data; 2) included data from cases with a diagnosis of schizophrenia determined using the Diagnostic and Statistical Manual or International Classification of Disease criteria; 3) included data from a healthy control group; 4) reported group measures for synaptophysin, SNAP-25, syntaxin, VAMP (also called synaptobrevin), complexin, synapsin, rab3a, synaptotagmin, or PSD-95; 5) published in a peer-reviewed journal; 6) written in English; 7) compared synaptophysin levels among groups.

Exclusion criteria were: 1) studies in patients with comorbid neurological disorders; 2) studies exclusively in animal models; 3) presented data which were not original (such as reviews); 4) studies which included the same postmortem brain material as other included studies (thus representing a duplicated subject) were excluded to avoid double counting; we did not exclude studies that used the same brains for studying different regions or different proteins; 5) did not provide data in a form that enabled group mean and/or variance to be determined, and the authors did not provide this information when requested ^1-3^. We took particular care in ascertaining no overlap existed between the samples used in different studies. When this happened, we excluded the smaller study. When this was not apparent on reading the articles we contacted the authors to establish this.

### DATA EXTRACTION:

Whenever sub-regional measures were reported, we collected these individually.

Data extraction was performed by one researcher (EFO) and the data were independently checked by a second author (KB).

Where data was presented as a plot rather than numerically, we used plot digitiser software [http://arohatgi.info/WebPlotDigitizer] to extract mean and variance measures. This software has been shown to be a reliable method of data extraction for meta-analyses ^4^. Wherever data were presented as quantiles ^5^, this was converted to mean and standard deviation using the method described in Wan et al. ^6^. One study indicated mean and standard deviation for patients with schizophrenia, but only the mean for controls ^7^; as a conservative estimate, we assigned the SD for controls equal to the double of that of patients. The average number of samples per study was 30. To be conservative, because some studies had sample sizes below 20 subjects, we used Hedges’ *g* as a measure of effect size. Hedges’ *g* is Cohen’s *d* multiplied by a correction factor that gives an unbiased estimation of *δ* for smaller samples ^8^.

### STATISTICAL ANALYSIS:

Heterogeneity between studies was measured using the I2 statistic, which describes the percentage of the variability in effect estimates that is due to heterogeneity, and tested using Cochrane’s Q-Test ^9^. Publication bias was assessed for each group of studies by visual inspection of funnel plots, and tested with a regression test for funnel plot asymmetry (mixed-effects meta-regression model). Where these were significant we conducted additional trim-and-fill analyses. p < 0.05 two tailed was considered statistically significant. Additional exploratory analyses were run for significant results to evaluate the effect of potential confounders (differences in postmortem interval and proportion with death due to suicide) with the same parameters as the main analysis and using random effects models. If sub-regions were reported, a dependent effect size was calculated, then averaged with the other sub regions of the same region ^10^, obtaining one single average effect size for each study for each region. For the analyses of data from the hippocampus, only data for hippocampal regions were used, whilst para-hippocampal regions were grouped together with other temporal areas. A p value of 0.05 (two-tailed) was set as the significance level. Meta-analyses were carried out using the *metafor* package (version 2.0 ^11^) in R 3.3 ^12^, using random effects (RE) models. When the main meta-analysis was significant, we conducted the following sensitivity analyses. When indicated, a trim and fill analysis was run to impute potentially missing studies. We also tested if the overall significance was affected excluding the studies where postmortem interval (PMI) was significantly different among cases and controls. Additional exploratory analyses were run for significant results to evaluate the potential effect of confounders. Both meta-regression (test for moderators) and sub-group analyses were performed in *metafor* with the same parameters as the main analysis (random effects models). When possible, we studied the fraction of the included population that had died by suicide and postmortem interval (PMI) as potential confounders because these are established factors that could affect postmortem biochemical analyses. We did not include age among the confounders we studied, as all of the studies we included were carefully matched for age of death. Illness duration was not studied as only a very small fraction of the studies contained this information.

## Supplementary Tables

### Supplementary Table 1: Summary of the studies of synaptophysin levels in schizophrenia: sample and methodological characteristics and findings

**Legend**:

- **Frontal cortex** to **occipital cortex** fields: graphic depiction of the results for these brain regions. **↔** = no significant change between cases and controls**. ↓** = significant reduction in patients with schizophrenia as compared to controls. ⇣ = absolute levels lower in schizophrenia but did not meet statistical significance on testing; **⇡** absolute levels greater in schizophrenia but did not meet statistical significance on testing
- **N SCZ, N controls**: number of samples per category, SCZ=schizophrenia
- **Technique**: WB = Western blotting, ELISA = enzyme-linked immunosorbent assay, IHC = Immunohistochemistry
- **Mean +-SD of AGE (years) SCZ/CTR**: patients' age +- Standard Deviation/Controls' age +- Standard Deviation
- **Reported SCZ Group Antipsychotic Treatment**: information available in the paper about treatment in the schizophrenia sample
- **Postmortem interval (scz/ctr) hours**: mean in hours for patients/controls (when available); NS = no statistically significant difference
- **Analysis blind to diagnosis?** did the authors report if the biochemical analyses were carried out by a scientist who was blind to sample diagnoses?
- SYN=synaptophysin levels, N/A= not available, RT-PCR= Reverse transcription polymerase chain reaction

| **authors** | **year** | **molecule** | **frontal cortex** | **hippo-campus** | **cingulate cortex** | **temporal cortex** | **occipital cortex** | **N, SCZ** | **N, controls** | **technique** | **Age** | **Antipsychotic Treatment in schizophrenia group** | **Mean (sd) postmortem interval (scz/ctr) hours; statistical significance** | **cause of death: number or proportion of suicides** | **analysis blind to diagnosis?** | **source of material** |
| --- | --- | --- | --- | --- | --- | --- | --- | --- | --- | --- | --- | --- | --- | --- | --- | --- |
| Eastwood et al | 1995  (May) | mRNA | n/a | **↓** | n/a | **↓** | n/a | 7 | 13 | In situ hybridisation | 60+-4/56+-5 | on typical neuroleptics for varying times | 42+-5/33+-3; NS | NA | Not specified | Dusseldorf-Oxford |
| Tcherepanov and Sokolow | 1997 | mRNA | n/a | n/a | n/a | **⇡** | n/a | 24 | 10 | RT-PCR | 74+-12/77+-11 | all on antipsychotics | 44+-38/5+-2; p<0.0001 | N/A | Not specified | Schizophrenia Brain Bank of the Department of Psychiatry at the Mount Sinai School of Medicine, New York |
| Karson et al | 1999 | mRNA | **↔** | n/a | n/a | n/a | n/a | 14 | 12 | Northern blot | 65+-12/67+-8 | 3 patients were not receiving antipsychotics at death | 10+-6/6+-4; NS | C: 0/12 S: 0/14 | Blind | N/A |
| Eastwood et al | 2000 | mRNA | **⇣** | n/a | n/a | **↓** | **↓** | 7-11 depending on brain region | 6-11 depending on brain region | IHC | 57+-5/63+-5 | all had received antipsychotics and all but two were on medication at death | 45+-5/39+-6; NS | N/A | Blind | London & Oxford Series |
| Glantz et al | 2000 | mRNA | **↔** | n/a | n/a | n/a | n/a | 10 | 10 | In situ hybridisation | 45+-10/48+-11 | 8 patients on antipsychotics at time of death | 18+-7/19+-4; NS | S: 1/10 suicide | Blind | Allegheny County Coroner’s Office |
| Webster et al | 2001 | mRNA | n/a | **⇣** | n/a | n/a | n/a | 11 | 10 | In situ hybridisation | 49/49 | N/A | 39/26; p = 0.1 | C: 0/10 S: 1/11 | Not specified | NIMH Neuroscience Center at Saint Elizabeth’s Hospital |
| Rao et al | 2013 | mRNA | **↓** | n/a | n/a | n/a | n/a | 10 | 10 | WB | 59±13.6/49±13.6 | One patient on trazodone, one on valproate, all others on antipsychotics | 22+-4.3/20±5.1; NS | no suicides | Blind | Harvard Brain Tissue Resource Center |
| Browning at al | 1993 | protein | n/a | **↔** | n/a | n/a | n/a | 7 | 7 | WB - immunoautoradiography | 61+-11/50+-19 | 6 on typical antipsychotics (n=1: missing information) | 16.2 +- 5.9 hr SD)/ 14.4 ± 6.2); NS | N/A | Not specified | Denver VA Medical Center, the Arapahoe County Coroner's Office, and National Disease Research Interchange, Philadelphia, PA |
| Eastwood et al | 1995 (Nov) | protein | n/a | **⇣** | n/a | **⇣** | n/a | 11 | 14 | IHC - immunoautoradiography | 57+-5/64+-4 | not specified | 45+-5/38+-5; NS | N/A | Not specified | Dusseldorf-Oxford + ? |
| Perrone-Bizzozero et al | 1996 | protein | **↓** | n/a | n/a | **⇣** | **⇣** | 13 - different for different areas | 13 - different for different areas | WB | average excluding area 17 pts: 47+-14/48+-15.9 | all on typical antipsychotics | 16+-7.3/15+-7.5; NS | C: 0/13 S: 4/13 | Blind | Brain Tissue Resource Center at the McLean Hospital (Belmont, MA) |
| Glantz and Lewis | 1997 | protein | **⇣** | n/a | n/a | n/a | **↔** | 10 | 10 | IHC | 55+-15/56+-14 | 8 on antipsychotic at death, 2 antipsychotic free | 11+-5.4/11+-6; NS | C: 0/10 S: 2/10 | Not specified | Allegheny County Coroner's Office, Pittsburgh, Pa, or University of Pittsburgh Medical Center. |
| Honer et al. | 1997 | protein | n/a | n/a | **↔** | n/a | n/a | 18 | 24 | ELISA | 49+-14.7/47+-19.1 | 7 antipsychotic drug in blood at death, 9 no antipsychotic in blood at death, 1 missing data, and 1 drug free for three months prior to death | 15+-9.6/18+-11.5; NS | N/A | Blind | N/A |
| Young et al | 1998 | protein | n/a | **⇣** | n/a | n/a | n/a | 13 | 13 | ELISA | 52+-17.8/45+-15.9 (ELISA) | N/A | ELISA sample: 26+- 23.3/23+-13.6; NS | C: 0/13 S: 5/13 | Blind | N/A |
| Davidsson et al | 1999 | protein | **⇣** | **↓** | **↓** | **↔** | n/a | 5-18 depending on brain region | 6-21 depending on brain region | WB | 81+-11.3/75+-12.8 | Yes but no details. Only reports on mean lifetime dose of neuroleptics | different for different brain areas, overall: NS | controls nil. Patients N/A | Not specified | Department of Psychiatry, Sahlgrenska University Hospital/Molndal, Sweden |
| Honer et al. | 1999 | protein | **↔** | n/a | n/a | n/a | n/a | 13 | 10 | ELISA | 48+-17.5/45+-16.9 | only 2 patients were on antipsychotics | 13.8+-6/18.4+-4.6; NS | C: 0/10 S: 6/13 | Blind | N/A |
| Karson et al | 1999 | protein | **↓** | n/a | n/a | n/a | n/a | 14 | 12 | WB | 65+-12/67+-8 | 3 patients were not receiving antipsychotics at death. | 10+-6/6+-4; NS | C: 0/12 S: 0/14 | Blind | N/A |
| Eastwood et al | 2000 | protein | **↔** | n/a | **↔** | **↔** | **⇣** | 7-11 depending on brain region | 6-11 depending on brain region | IHC - immunoautoradiography | 57+-5/63+-5 | All had received antipsychotics and all but two were on medication at death | 45+-5/39+-6; NS | N/A | Blind | London & Oxford Series |
| Eastwood and Harrison | 2001 | protein | n/a | n/a | **⇣** | n/a | n/a | 15 | 14 | WB | 45+-13/47+-9.5 | 12 on antipsychotics at death, 14 had lifetime antipsychotic | 34+-14.6/25+-9.8; NS. | suicides: 4/15 in scz, nil controls | Blind | Stanley Foundation Neuropathology Consortium, MD, USA |
| Landén et al | 2002 | protein | n/a | n/a | **↓** | n/a | n/a | 11 | 13 | WB | 80+-10/75+-14 | 3 drug free, others on antipsychotics | 40+-14/75+-47; p<0.05 | N/A | Not specified | N/A |
| Vawter et al | 2002 | protein | n/a | **↔** | n/a | n/a | n/a | 16 | 13 | WB | 52+-16.4/52+-17.3 | N/A | 24+-21.6/23+-11.5; NS | C: 0/13 S: 4/16 | Blind | Washington, DC Medical Examiners Office |
| Halim et al | 2003 | protein | **↔** | n/a | n/a | n/a | n/a | 18 | 23 | WB | 51+-16/49+-16 | Yes but no details. Only reports on mean lifetime dose of neuroleptics | 32+-17/27+-15; NS | C: 0/23 S: 2/18 | Not specified | Clinical Brain Disorders Branch (Bethesda, MD, USA) |
| Talbot et al | 2004 | protein | n/a | **⇣** | n/a | n/a | n/a | 17 | 17 | IHC | 44/48 | unclear | 34/24 NS | N/A | Blind | Stanley Foundation Neuropathology Consortium, MD, USA |
| Chambers et al | 2005 | protein | n/a | **↓** | n/a | n/a | n/a | 14 | 14 | IHC | 45±11/47+-14 | 13 on antipsychotics (6 atypical, 5 typical, 2 both) 1 non medicated | 16±5 h/19±3 h; NS | C: 0 suicides (2 NA) S: 2 suicides (3 N/A) | Blind | Harvard Brain Tissue Resource Center at McLean Hospital, Belmont, MA |
| Beasley et al | 2005 | protein | n/a | n/a | n/a | n/a | **⇣** | 15 | 15 | ELISA | 44+-13/48+-11 | NA | 34+-15/24+-10, p=0.04 | C: 0/15 S: 4/15 | Not specified | Stanley Foundation Neuropathology Consortium, MD, USA |
| Scarr et al | 2006 | protein | **⇣** | n/a | n/a | n/a | n/a | 20 | 20 | WB | 56+-13/56+-12.6 | Yes but no details. Only reports on mean lifetime dose of neuroleptics | 43+-13.4/39+-15.8; NS | N/A | Not specified | CNS repository at the Rebecca L. Cooper Research Laboratories |
| Gray et al | 2010 | protein | **↔** | n/a | n/a | n/a | n/a | 20 | 20 | WB | 56+-13/56+-12.6 | all on antipsychotics | 43+-8.9/39+-11.3; NS | C: 0/20 S: 4/20 | Not specified | Rebecca L. Cooper Research Laboratories post-mortem CNS repository. |
| Glantz et al | 2010 | protein | n/a | n/a | n/a | **⇣** | n/a | 14 | 15 | WB | 45+-13/48+-11 | NA | 34+-15/24+-10, p=0.04 | NA | Blind | Stanley Foundation Neuropathology Consortium, MD, USA |
| Gilabert-Juan et al | 2012 | protein | **↔** | n/a | n/a | n/a | n/a | 15 | 15 | IHC | 45+-13.2/48+-10.8 | not specified | 34+-14.7/24+-10; p<0.05 | suicides: 4 in scz, nil controls | Not specified | Stanley Medical Research Institute (Bethesda,  MD, USA)` |
| Gil-Pisa et al | 2012 | protein | **↔** | n/a | n/a | n/a | n/a | 24 | 24 | WB | 39+-3/39+-2 | 12 treated, 12 antipsychotic free at death | 23+-3/23+-3; NS | C: 0/24 S: 19/24 | Not specified | Basque Institute of Legal Medicine, Bilbao (Spain), and Romand University Center of Legal Medicine – Geneva |
| Rao et al | 2013 | protein | **↓** | n/a | n/a | n/a | n/a | 10 | 10 | WB | 59±13.6/49±13.6 | One patient on trazodone, one on valproate, all others on antipsychotics | 22+-4.3/20±5.1; NS | no suicides | Blind | Harvard Brain Tissue Resource Center |
| Barksdale et al | 2014 | protein | n/a | n/a | **↔** | n/a | n/a | 25 | 13 | WB | 43+-12/37+-10 | All on typical or atypical antipsychotics (groups analysed separately: no difference in SYN) | 14/14.5; NS | N/A | Blind | Maryland Brain Collection |
| Föcking et al | 2015 | protein | n/a | n/a | **↔** | n/a | n/a | 20 | 20 | WB | 42+-8.8/44+-8.0 | N/A | 23+-8.0/21+-7.3; NS | C: 0/20 S: 4/20 | Blind | Stanley Medical Research Institute |
| Matosin N et al | 2016 | protein | n/a | **↓** | n/a | n/a | n/a | 20 | 20 | WB | 56±13.5/58±12.6 | Yes but no details. Only reports on mean lifetime dose of neuroleptics | 28+-10.1/26+-12.8; NS | C: 0/20 S: 4/20 | Blind | NSW Brain Tissue Resource Centre (Sydney, NSW, Australia) |

### Supplementary Table 2: Summary of the studies of SNAP-25 levels in schizophrenia: sample and methodological characteristics and findings

**Legend**:

- **Frontal cortex** to **temporal cortex** fields: graphic depiction of the results for these brain regions. **↔** = no significant change between cases and controls**. ↓** = significant reduction in patients with schizophrenia as compared to controls. ⇣ = absolute levels lower in schizophrenia but did not meet statistical significance on testing; **⇡** absolute levels greater in schizophrenia but did not meet statistical significance on testing
- **N SCZ, N controls**: number of samples per category, SCZ=schizophrenia
- **Technique**: WB = Western blotting, ELISA = enzyme-linked immunosorbent assay, IHC = Immunohistochemistry
- **Mean +-SD of AGE (years) SCZ/CTR**: patients' age +- Standard Deviation/Controls' age +- Standard Deviation
- **Reported SCZ Group Antipsychotic Treatment**: information available in the paper about treatment in the schizophrenia sample
- **Postmortem interval (scz/ctr) hours**: mean in hours for patients/controls (when available); NS = no statistically significant difference
- **Analysis blind to diagnosis?** did the authors report if the biochemical analyses were carried out by a scientist who was blind to sample diagnoses?
- N/A= not available, RT-PCR= Reverse transcription polymerase chain reaction

| **authors** | **year** | **molecule** | **frontal cortex** | **hippocampus** | **Cingulate cortex** | **temporal cortex** | **N, SCZ** | **N, controls** | **technique** | **Age** | **Antipsychotic Treatment in schizophrenia group** | **Mean (sd) postmortem interval (scz/ctr) hours; statistical significance** | **cause of death: number or proportion of suicides** | **analysis blind to diagnosis?** | **source of material** |
| --- | --- | --- | --- | --- | --- | --- | --- | --- | --- | --- | --- | --- | --- | --- | --- |
| Karson et al | 1999 | mRNA | **↔** | n/a | n/a | n/a | 14 | 12 | Northern blot | 65+-12/67+-8 | 3 patients were not on antipsychotics at death | 10+-6/6+-4; NS | C: 0/12 S: 0/14 | Blind | N/A |
| Sokolov et al | 2000 | mRNA | n/a | n/a | n/a | **↔** | 14 | 9 | RT-PCR | 77+-3/77+-4 | 4 pts on treatment at time of death | 41+-11/5+-1 | no suicides | Blind | Schizophrenia Brain Bank of the Department of Psychiatry at the Mount Sinai School of Medicine, New York |
| Scarr et al | 2006 | mRNA | **⇣** | n/a | n/a | n/a | 18 | 18 | RT-PCR | 56+-13/56+-12.6 | Yes. Reports mean lifetime dose of neuroleptics | 43+-13.4/39+-15.8; NS | N/A | Not specified | CNS repository at the Rebecca L. Cooper Research Laboratories |
| Thompson et al | 2015 | mRNA | **⇣** | n/a | n/a | n/a | 8 | 12 | RT-PCR | 50+-11/56+-16 | 3 not on antipsychotics at time of death | 30+-4.6/26+-4.7; NS | C: 0/12 S: 1/8 | Not specified | Southwest Brain Bank (SWBB) collection of postmortem tissue for research (USA) |
| Honer et al. | 1997 | protein | n/a | n/a | **⇡** | n/a | 18 | 24 | ELISA | 49+-14.7/47+-19.1 | 7 antipsychotic drug in blood at death, 9 no antipsychotic in blood at death, 1 missing data, and 1 drug free for three months prior to death | 15+-9.6/18+-11.5; NS | N/A | Blind | N/A |
| Thompson et al | 1998 | protein | **↔** | n/a | n/a | **↓** | 25 (different in different tissues) | 31 (different in different tissues) | WB | average excluding area 17 pts: 55+-15.8/51+-17.8 | All on neuroleptics, 2 also on lithium | 16+-9.3/13+-8; NS | C: 1/31 S: 4/25 | Not specified | Brain Tissue Resource Center at  the McLean Hospital, Belmont, Massachusetts |
| Young et al | 1998 | protein | n/a | **⇣** | n/a | n/a | 13 | 13 | ELISA | 52+-17.8/45+-15.9 (ELISA) | N/A | ELISA sample: 26+- 23.3/23+-13.6; NS | C: 0/13 S: 5/13 | Blind | N/A |
| Karson et al | 1999 | protein | **↓** | n/a | n/a | n/a | 14 | 12 | WB | 65+-12/67+-8 | 3 patients were not receiving antipsychotics at death. | 10+-6/6+-4; NS | C: 0/12 S: 0/14 | Blind | N/A |
| Fatemi et al | 2001 | protein | n/a | **↔** | n/a | **⇣** | 15 | 15 | IHC | 44/48 | 10 out of 15 were on antipsychotics | 33.7/23.7; NS | C:0/15 S: 4/15 | Blind | Stanley Foundation Neuropathology Consortium, MD, USA |
| Honer et al. | 2002 | protein | **⇣** | n/a | n/a | n/a | 13 | 11 | ELISA | 48+-17.5/46+-17 | Antipsychotic drugs were detected in two cases, and antidepressant drugs in three. All likely to have been previously prescribed antipsychotic drugs. | 13.8+-6/18.4+-4.6; NS | C: 0/11 S: 7/13 | Blind | Clinical Brain Disorder Branch NIMH Washington + Author's brain bank |
| Halim et al | 2003 | protein | **↔** | n/a | n/a | n/a | 18 | 23 | WB | 51+-16/49+-16 | Yes. Reports mean lifetime dose of neuroleptics | 32+-17/27+-15; NS | C: 0/23 S: 2/18 | Not specified | Clinical Brain Disorders Branch (Bethesda, MD, USA) |
| Thompson et al | 2003 | protein | n/a | **↓** | n/a | n/a | 7 | 8 | Western blot. | 52+-19.3/49+-13.6 | 2 on neuroleptics | 12.5+-8.2/25+-13.8; significantly different | N/A | Not specified | NIMH Neuroscience Center at Saint Elizabeth’s Hospital |
| Scarr et al | 2006 | protein | **↔** | n/a | n/a | n/a | 20 | 20 | WB | 56+-13/56+-12.6 | Yes. Reports mean lifetime dose of neuroleptics | 43+-13.4/39+-15.8; NS | N/A | Not specified | CNS repository at the Rebecca L. Cooper Research Laboratories |
| Gray et al | 2010 | protein | **↔** | n/a | n/a | n/a | 20 | 20 | WB | 56+-13/56+-12.6 | all on antipsychotics | 43+-8.9/39+-11.3; NS | C: 0/20 S: 4/20 | Not specified | Rebecca L. Cooper Research Laboratories post-mortem CNS repository. |
| Gil-Pisa et al | 2012 | protein | **↔** | n/a | n/a | n/a | 24 | 24 | WB | 39+-3/39+-2 | 12 treated, 12 antipsychotic free at death | 23+-3/23+-3; NS | C: 0/24 S: 19/24 | Not specified | Basque Institute of Legal Medicine, Bilbao (Spain), and Romand University Center of Legal Medicine – Geneva |
| Thompson et al | 2015 | protein | **↔** | n/a | **↔** | n/a | 8 | 12 | WB | 50+-11/56+-16 | 3 not on antipsychotics at time of death | 30+-4.6/26+-4.7; NS | C: 0/12 S: 1/8 | Not specified | Southwest Brain Bank (SWBB) collection of postmortem tissue for research (USA) |
| Ramos-Miguel et al | 2015 | protein | **↔** | n/a | n/a | n/a | 15 | 13 | WB | Cohort 1: 54 ± 12.1/51 ± 18.8 | Cohort 1: 2 on antipsychotics at death | Cohort 1: 9+-3.4/17+-7.1 | C: 4/9/0 S: 12/3/0 | Blind | Macedonian/New York State Psychiatric Institute Brain Collection |

### Supplementary Table 3: Summary of the studies of PSD-95 levels in schizophrenia: sample and methodological characteristics and findings

**Legend**:

- **Frontal cortex** to **temporal cortex** fields: graphic depiction of the results for these brain regions. **↔** = no significant change between cases and controls**. ↓** = significant reduction in patients with schizophrenia as compared to controls. ⇣ = absolute levels lower in schizophrenia but did not meet statistical significance on testing; **⇡** absolute levels greater in schizophrenia but did not meet statistical significance on testing
- **N SCZ, N controls**: number of samples per category, SCZ=schizophrenia
- **Technique**: WB = Western blotting, ELISA = enzyme-linked immunosorbent assay, IHC = Immunohistochemistry
- **Mean +-SD of AGE (years) SCZ/CTR**: patients' age +- Standard Deviation/Controls' age +- Standard Deviation
- **Reported SCZ Group Antipsychotic Treatment**: information available in the paper about treatment in the schizophrenia sample
- **Postmortem interval (scz/ctr) hours**: mean in hours for patients/controls (when available); NS = no statistically significant difference
- **Analysis blind to diagnosis?** did the authors report if the biochemical analyses were carried out by a scientist who was blind to sample diagnoses?
- N/A= not available, RT-PCR= Reverse transcription polymerase chain reaction

| **authors** | **year** | **molecule** | **frontal cortex** | **hippocampus** | **ACC** | **occipital cortex** | **N, SCZ** | **N, controls** | **technique** | **Age** | **Antipsychotic Treatment in schizophrenia group** | **Mean (sd) postmortem interval (scz/ctr) hours; statistical significance** | **cause of death: number or proportion of suicides** | **analysis blind to diagnosis?** | **source of material** |
| --- | --- | --- | --- | --- | --- | --- | --- | --- | --- | --- | --- | --- | --- | --- | --- |
| Dracheva et al | 2001 | mRNA | **⇡** | n/a | n/a | **↑** | 26 | 13 | RT-PCR | 72+-12/83+-10; p=0.01 | 9/26 not on antipsychotics at time of death | 15+-9/8+-6; p=0.03 | N/A | N/A | Brain Bank, Department of Psychiatry, Mount Sinai/Bronx Veterans Administration Medical Center. |
| Kristiansen et al | 2006 | mRNA | **↔** | n/a | **↑** | n/a | 18 | 11 | In situ hybridisation | 80+- 11; p = 0.072 | N/A | 10+-6; p = 0.275 | N/A | N/A | Mount Sinai Medical Center Brain Bank |
| Ide and Lewis | 2010 | mRNA | **↔** | n/a | n/a | n/a | 31 | 31 | RT-PCR | 47+-12/47+-12 | NA | 18+-9/17+-6; NS | N/A | N/A | Allegheny County Medical Examiner’s Office |
| Funk et al | 2017 | mRNA | **⇣** | n/a | n/a | n/a | 175 | 210 | qPCR (Taqman) | 50+-15/44+-16; p=0.0002 | N/A | 39+-24/31+-15; p<0.0001 | N/A | N/A | Human Brain Collection Core (HBCC), NIMH |
| Toyooka et al | 2002 | protein | **↔** | **↔** | n/a | **↔** | 13-21 depending on area | 16-25 depending on area | WB | 62+-15/61+-13 | all on long term typical antipsychotics | variable for different samples. NS difference for frontal and occipital, significant difference in hippocampus | S: 0/21 | N/A | Matsuzawa Hospital |
| Toro and Deakin | 2005 | protein | **↔** | **⇣** | n/a | n/a | 15 | 15 | IHC | 45+-13/48+-11 | all on antipsychotics | 34+-15/24+-10; p=0.6 | C: 0/15 S: 4/15 | Blind | Stanley Foundation Neuropathology Consortium, MD, USA |
| Hahn et al | 2006 | protein | **↔** | n/a | n/a | n/a | 14 | 14 | WB | 79+-7/78+-9 | 6 patients not on antipsychotics | 11+-7/11+-3; NS | N/A | N/A | Prospective clinicopathological studies program at the University of Pennsylvania |
| Kristiansen et al | 2006 | protein | **↔** | n/a | **↓** | n/a | 13 | 8 | WB | 75+-12; p = 0.134 | N/A | 8+-5; p = 0.072 | N/A | N/A | Mount Sinai Medical Center Brain Bank |
| Funk et al | 2012 | protein | **↔** | n/a | **↓** | n/a | 35-36 depending on area | 31-33 depending on area | WB | 74+-12/78+-14 | 11 were antipsychotic free at death | 13+-7/8+-7; p=0.005 | no suicides | N/A | Mount Sinai Medical Center Brain Bank |
| Catts et al | 2015 | protein | **↓** | n/a | n/a | n/a | 37 | 37 | WB | 51+-14/51+-15 | all on antipsychotics | 28+-14/25+-11; p=0.2 | C: 0/37 S: 8/37 | Blind | New South  Wales Tissue Resource Centre collection |
| Föcking et al | 2015 | protein | n/a | n/a | **↔** | n/a | 20 | 20 | unbiased shotgun proteomics | 42+-8.8/44+-8.0 | N/A | 23+-8.0/21+-7.3; NS | C: 0/20 S: 4/20 | Blind | Stanley Medical  Research Institute’s (SMRI) Array Collection |
| Matosin N et al | 2016 | protein | n/a | **↓** | n/a | n/a | 20 | 20 | WB | 56±13.5/58±12.6 | Yes. Reports mean lifetime dose of neuroleptics | 28+-10.1/26+-12.8; NS | C: 0/20 S: 4/20 | Blind | NSW Brain Tissue Resource Centre (Sydney, NSW, Australia) |
| Funk et al | 2017 | protein | **↓** | n/a | n/a | n/a | 26 | 47 | WB | 59+-17/42+-15; p < 0.0001 | N/A | 42+-22/35+-16; p=0.11 | N/A | N/A | Human Brain Collection Core (HBCC), NIMH |

### Supplementary Table 4: Summary of the studies of syntaxin levels in schizophrenia: sample and methodological characteristics and findings

**Legend**:

- **Frontal cortex** to **temporal cortex** fields: graphic depiction of the results for these brain regions. **↔** = no significant change between cases and controls**. ↓** = significant reduction in patients with schizophrenia as compared to controls. ⇣ = absolute levels lower in schizophrenia but did not meet statistical significance on testing; **⇡** absolute levels greater in schizophrenia but did not meet statistical significance on testing
- **N SCZ, N controls**: number of samples per category, SCZ=schizophrenia
- **Technique**: WB = Western blotting, ELISA = enzyme-linked immunosorbent assay, IHC = Immunohistochemistry
- **Mean +-SD of AGE (years) SCZ/CTR**: patients' age +- Standard Deviation/Controls' age +- Standard Deviation
- **Reported SCZ Group Antipsychotic Treatment**: information available in the paper about treatment in the schizophrenia sample
- **Postmortem interval (scz/ctr) hours**: mean in hours for patients/controls (when available); NS = no statistically significant difference
- **Analysis blind to diagnosis?** did the authors report if the biochemical analyses were carried out by a scientist who was blind to sample diagnoses?
- N/A= not available, RT-PCR= Reverse transcription polymerase chain reaction

| **authors** | **year** | **molecule** | **frontal cortex** | **temporal c** | **N, SCZ** | **N, controls** | **technique** | **Age** | **Antipsychotic Treatment in schizophrenia group** | **Mean (sd) postmortem interval (scz/ctr) hours; statistical significance** | **cause of death: number or proportion of suicides** | **analysis blind to diagnosis?** | **source of material** |
| --- | --- | --- | --- | --- | --- | --- | --- | --- | --- | --- | --- | --- | --- |
| Sokolov et al | 2000 | mRNA | n/a | **↔** | 14 | 9 | RT-PCR | 77+-3/77+-4 | 4 pts on treatment at time of death | 41+-11/5+-1; p<0.05 | no suicides | Blind | Schizophrenia Brain Bank of the Department of Psychiatry at the Mount Sinai School of Medicine, New York |
| Honer et al. | 2002 | protein | **↔** | n/a | 13 | 11 | ELISA | 48+-17.5/46+-17 | Antipsychotic drugs were detected in two cases, and antidepressant drugs in three. All the cases with schizophrenia were likely to have been previously prescribed antipsychotic drugs. | 13.8+-6/18.4+-4.6; NS | C: 0/11 S: 7/13 | Blind | Clinical Brain Disorder Branch NIMH Washington + Author's brain bank |
| Halim et al | 2003 | protein | **↔** | n/a | 18 | 23 | WB | 51+-16/49+-16 | Yes. Reports mean lifetime dose of neuroleptics | 32+-17/27+-15; NS | C: 0/23 S: 2/18 | Not specified | Clinical Brain Disorders Branch (Bethesda, MD, USA) |
| Scarr et al | 2006 | protein | **↔** | n/a | 20 | 20 | WB | 56+-13/56+-12.6 | Yes. Reports mean lifetime dose of neuroleptics | 43+-13.4/39+-15.8; NS | N/A | Not specified | CNS repository at the Rebecca L. Cooper Research Laboratories |
| Gray et al | 2010 | protein | **↔** | n/a | 20 | 20 | WB | 56+-13/56+-12.6 | all on antipsychotics | 43+-8.9/39+-11.3; NS | C: 0/20 S: 4/20 | Not specified | Rebecca L. Cooper Research Laboratories post-mortem CNS repository. |
| Castillo et al | 2010 | protein | **↔** | n/a | 15 | 15 | WB | 49+-18/50+-16 | N/A | 20+-8/17+-8; NS | N/A | Not specified | Dallas Brain Collection |
| Gil-Pisa et al | 2012 | protein | **↑** | n/a | 24 | 24 | WB | 39+-3/39+-2 | 12 treated, 12 antipsychotic free at death | 23+-3/23+-3; NS | C: 0/24 S: 19/24 | Not specified | Basque Institute of Legal Medicine, Bilbao (Spain), and Romand University Center of Legal Medicine – Geneva |
| Ramos-Miguel et al | 2015 | protein | **↔** | n/a | 15 | 13 | WB | Cohort 1: 54 ± 12.1/51 ± 18.8 | Cohort 1: 2 on antipsychotics at death | Cohort 1: 9+-3.4/17+-7.1; p=0.0006 | C: 4/9/0 S: 12/3/0 | Blind | Macedonian/New York State Psychiatric Institute Brain Collection |

### Supplementary Table 5: Summary of the studies of VAMP (synaptobrevin) levels in schizophrenia: sample and methodological characteristics and findings

**Legend**:

- **Frontal cortex** to **temporal cortex** fields: graphic depiction of the results for these brain regions. **↔** = no significant change between cases and controls**. ↓** = significant reduction in patients with schizophrenia as compared to controls. ⇣ = absolute levels lower in schizophrenia but did not meet statistical significance on testing; **⇡** absolute levels greater in schizophrenia but did not meet statistical significance on testing
- **N SCZ, N controls**: number of samples per category, SCZ=schizophrenia
- **Technique**: WB = Western blotting, ELISA = enzyme-linked immunosorbent assay, IHC = Immunohistochemistry
- **Mean +-SD of AGE (years) SCZ/CTR**: patients' age +- Standard Deviation/Controls' age +- Standard Deviation
- **Reported SCZ Group Antipsychotic Treatment**: information available in the paper about treatment in the schizophrenia sample
- **Postmortem interval (scz/ctr) hours**: mean in hours for patients/controls (when available); NS = no statistically significant difference
- **Analysis blind to diagnosis?** did the authors report if the biochemical analyses were carried out by a scientist who was blind to sample diagnoses?
- N/A= not available, RT-PCR= Reverse transcription polymerase chain reaction

| **authors** | **year** | **molecule** | **frontal cortex** | **temporal c** | **N, SCZ** | **N, controls** | **technique** | **Age** | **Antipsychotic Treatment in schizophrenia group** | **Mean (sd) postmortem interval (scz/ctr) hours; statistical significance** | **cause of death: number or proportion of suicides** | **analysis blind to diagnosis?** | **source of material** |
| --- | --- | --- | --- | --- | --- | --- | --- | --- | --- | --- | --- | --- | --- |
| Sokolov et al | 2000 | mRNA | n/a | **↔** | 14 | 9 | RT-PCR | 77+-3/77+-4 | 4 pts on treatment at time of death | 41+-11/5+-1; p<0.05 | no suicides | Blind | Schizophrenia Brain Bank of the Department of Psychiatry at the Mount Sinai School of Medicine, New York |
| Fung et al | 2011 | mRNA | **↔** | n/a | 37 | 37 | RT-PCR | 51/51 | all on antipsychotics | 29+-14/25+-11; NS | C: 0/37 S: 8/37 | Not specified | New South Wales Tissue Resource  Centre (Sydney, Australia |
| Honer et al | 2002 | protein | **↔** | n/a | 13 | 11 | ELISA | 48+-17.5/46+-17 | Antipsychotic drugs were detected in two cases, and antidepressant drugs in three | 13.8+-6/18.4+-4.6; NS | C: 0/11 S: 7/13 | Blind | Clinical Brain Disorder Branch NIMH Washington + Author's brain bank |
| Halim et al | 2003 | protein | **↓** | n/a | 18 | 23 | WB | 51+-16/49+-16 | Yes. Reports mean lifetime dose of neuroleptics | 32+-17/27+-15; NS | C: 0/23 S: 2/18 | Not specified | Clinical Brain Disorders Branch (Bethesda, MD, USA) |
| Scarr et al | 2006 | protein | **⇣** | n/a | 20 | 20 | WB | 56+-13/56+-12.6 | Yes. Reports mean lifetime dose of neuroleptics | 43+-13.4/39+-15.8; NS | N/A | Not specified | CNS repository at the Rebecca L. Cooper Research Laboratories |
| Gray et al | 2010 | protein | **↔** | n/a | 20 | 20 | WB | 56+-13/56+-12.6 | all on antipsychotics | 43+-8.9/39+-11.3; NS | C: 0/20 S: 4/20 | Not specified | Rebecca L. Cooper Research Laboratories post-mortem CNS repository. |
| Gil-Pisa et al | 2012 | protein | **⇣** | n/a | 24 | 24 | WB | 39+-3/39+-2 | 12 treated, 12 antipsychotic free at death | 23+-3/23+-3; NS | C: 0/24 S: 19/24 | Not specified | Basque Institute of Legal Medicine, Bilbao (Spain), and Romand University Center of Legal Medicine – Geneva |
| Ramos-Miguel et al | 2015 | protein | **↔** | n/a | 15 | 13 | WB | Cohort 1: 54 ± 12.1/51 ± 18.8 | Cohort 1: 2 on antipsychotics at death | Cohort 1: 9+-3.4/17+-7.1; p<0.001 | C: 4/9/0 S: 12/3/0 | Blind | Macedonian/New York State Psychiatric Institute Brain Collection |

### Supplementary Table 6: Summary of the studies of complexin levels in schizophrenia: sample and methodological characteristics and findings

**Legend**:

- **Frontal cortex** to **temporal cortex** fields: graphic depiction of the results for these brain regions. **↔** = no significant change between cases and controls**. ↓** = significant reduction in patients with schizophrenia as compared to controls. ⇣ = absolute levels lower in schizophrenia but did not meet statistical significance on testing; **⇡** absolute levels greater in schizophrenia but did not meet statistical significance on testing
- **N SCZ, N controls**: number of samples per category, SCZ=schizophrenia
- **Technique**: WB = Western blotting, ELISA = enzyme-linked immunosorbent assay, IHC = Immunohistochemistry
- **Mean +-SD of AGE (years) SCZ/CTR**: patients' age +- Standard Deviation/Controls' age +- Standard Deviation
- **Reported SCZ Group Antipsychotic Treatment**: information available in the paper about treatment in the schizophrenia sample
- **Postmortem interval (scz/ctr) hours**: mean in hours for patients/controls (when available); NS = no statistically significant difference
- **Analysis blind to diagnosis?** did the authors report if the biochemical analyses were carried out by a scientist who was blind to sample diagnoses?
- Cx I and Cx II = Complexin I and II, N/A= not available, RT-PCR= Reverse transcription polymerase chain reaction

| **authors** | **year** | **molecule** | **frontal cortex** | **hippocampus** | **Cingulate cortex** | **temporal cortex** | **N, SCZ** | **N, controls** | **technique** | **Age** | **Antipsychotic Treatment in schizophrenia group** | **Mean (sd) postmortem interval (scz/ctr) hours; statistical significance** | **cause of death: number or proportion of suicides** | **analysis blind to diagnosis?** | **source of material** |
| --- | --- | --- | --- | --- | --- | --- | --- | --- | --- | --- | --- | --- | --- | --- | --- |
| Harrison & Eastwood | 1998 | mRNA | n/a | Cx I ↔ - Cx II ↓ | n/a | Cx I ↔ - Cx II ↓ | 11 | 11 | immunoautoradiography | 57+-5/63+-5 | They had received antipsychotic drugs and all but one were on medication at the time of death. | 45+-5/37+-4; p= 0.0005 | N/A | Not specified | N/A |
| Eastwood and Harrison | 2000 | mRNA | n/a | Cx I ⇣ - Cx II ⇣ | n/a | Cx I ⇣ - Cx II ⇣ | 15 | 15 | IHC | 45+-3/49+-3 | 12/15 on antipsychotics | p=0.0385 | suicide 0 in ctr, 4/15 in scz | Blind | London & Oxford Series |
| Eastwood and Harrison | 2005 | mRNA | Cx I ↔ - Cx II ↓ | n/a | n/a | Cx I ↔ - Cx II ↓ | 9-10 depending on areas | 9 | IHC | 55+-19/50+-18 | all prescribed antipsychotic medication at death | 44+-18/36+-14; NS | C: 0/18 S: 2/13 | Not specified | N/A |
| Fung et al | 2011 | mRNA | Cx I ↔ - Cx II ↔ | n/a | n/a | n/a | 37 | 37 | RT-PCR | 51/51 | all on antipsychotics | 29+-14/25+-11; NS | C: 0/37 S: 8/37 | Not specified | New South Wales Tissue Resource  Centre (Sydney, Australia |
| Harrison & Eastwood | 1998 | protein | n/a | Cx I ↔ - Cx II ⇣ | n/a | Cx I ↔ - Cx II ↓ | 11 | 11 | immunoautoradiography | 57+-5/63+-5 | They had received antipsychotic drugs and all but one were on medication at the time of death. | 45+-5/37+-4; p=0.0005 | N/A | Not specified | N/A |
| Eastwood and Harrison | 2001 | protein | n/a | n/a | Cx I ↔ - Cx II ⇣ | n/a | 15 | 14 | WB | 45+-13/47+-9.5 | 12 on antipsychotics at death, 14 had lifetime antipsychotic | 34+-14.6/25+-9.8; NS | suicides: 4/15 in scz, nil controls | Blind | Stanley Foundation Neuropathology Consortium, MD, USA |
| Sawada | 2002 | protein | Cx I ↓ - Cx II ↔ | n/a | n/a | n/a | 13 | 11 | ELISA | 48+-17.4/46+-17.0 | Antipsychotic drugs were detected in two cases, and antidepressant drugs in three. All likely to have been previously prescribed antipsychotic drugs | 14+-6.0/18+-4.8; NS | C: 8/3/0 S: 5/2/6 | Blind | N/A |
| Sawada | 2005 | protein | n/a | ↓ | n/a | n/a | 12 | 12 | IHC | 57+-9.4/54+-16.8 | 9 haloperidol, 1 pericyazine, 1 Methotrimeprazine, 1 NA | 9+-6.7/14+-5.3; NS | C: 11/1/0 S: 9/0/0+3NA | Not specified | N/A |
| Ramos-Miguel et al | 2015 | protein | ↔ | n/a | n/a | n/a | 15 | 13 | WB | Cohort 1: 54 ± 12.1/51 ± 18.8 | Cohort 1: 2 on antipsychotics at death | Cohort 1: 9+-3.4/17+-7.1; p<0.001 | C: 4/9/0 S: 12/3/0 | Blind | Macedonian/New York State Psychiatric Institute Brain Collection |

### Supplementary Table 7: Summary of the studies of synapsins levels in schizophrenia: sample and methodological characteristics and findings

**Legend**:

- **Frontal cortex** to **temporal cortex** fields: graphic depiction of the results for these brain regions. **↔** = no significant change between cases and controls**. ↓** = significant reduction in patients with schizophrenia as compared to controls. ⇣ = absolute levels lower in schizophrenia but did not meet statistical significance on testing; **⇡** absolute levels greater in schizophrenia but did not meet statistical significance on testing
- **N SCZ, N controls**: number of samples per category, SCZ=schizophrenia
- **Technique**: WB = Western blotting, ELISA = enzyme-linked immunosorbent assay, IHC = Immunohistochemistry
- **Mean +-SD of AGE (years) SCZ/CTR**: patients' age +- Standard Deviation/Controls' age +- Standard Deviation
- **Reported SCZ Group Antipsychotic Treatment**: information available in the paper about treatment in the schizophrenia sample
- **Postmortem interval (scz/ctr) hours**: mean in hours for patients/controls (when available); NS = no statistically significant difference
- **Analysis blind to diagnosis?** did the authors report if the biochemical analyses were carried out by a scientist who was blind to sample diagnoses?
- Sy I and Sy II = synapsin I and II, N/A= not available, RT-PCR= Reverse transcription polymerase chain reaction

| **authors** | **molecule** | **year** | **frontal cortex** | **hippocampus** | **temporal** | **N, SCZ** | **N, controls** | **technique** | **Age** | **Antipsychotic Treatment in schizophrenia group** | **Mean (sd) postmortem interval (scz/ctr) hours; statistical significance** | **cause of death: number or proportion of suicides** | **analysis blind to diagnosis?** | **source of material** |
| --- | --- | --- | --- | --- | --- | --- | --- | --- | --- | --- | --- | --- | --- | --- |
| Tcherepanov and Sokolow | mRNA | 1997 | n/a | n/a | Sy I ⇡ | 24 | 10 | RT-PCR | 74+-12/77+-11 | all on antipsychotics | 44+-38/5+-2; p<0.0001 | N/A | Not specified | Schizophrenia Brain Bank of the Department of Psychiatry at the Mount Sinai School of Medicine, New York |
| Imai et al | mRNA | 2001 | Sy II ↔ | n/a | n/a | 6 | 8 | RT-QPCR | 60+-10.7/58+-13.3 | not specified | 8+-4.5/6+-4.1; NS | C: 8/0/0 S: 4/2/0 | Not specified | N/A |
| Tan et al | mRNA | 2014 | Sy II ↓ | n/a | n/a | 35 | 35 | RT-PCR | 43+-9/44+-8 | Yes. Reports mean lifetime dose of neuroleptics | 31+-16/29+-13; p=0.6 | N/A | Blind | Stanley Array Collection of the Stanley Foundation Brain Collection |
| Browning at al | protein | 1993 | n/a | Sy I: ↓ Sy IIb ⇣ | n/a | 25 | 13 | WB | 43+-12/37+-10 | All on typical or atypical antipsychotics | 14/14.5; NS | N/A | Blind | Denver VA Medical Center, the Arapahoe County Coroner's Office, and National Disease Research Interchange, Philadelphia, PA |
| Imai et al | protein | 2001 | Sy II ↔ | n/a | n/a | 6 | 8 | WB | 60+-10.7/58+-13.3 | N/A | 8+-4.5/6+-4.1; NS | C: 8/0/0 S: 4/2/0 | Not specified | N/A |
| Vawter MP et al | protein | 2002 | n/a | Tot: ↓ Sy I ↔ | n/a | 16 | 13 | WB | 52+-16.4/52+-17.3 | N/A | 24+-21.6/23+-11.5; NS | C: 0/13 S: 4/16 | Blind | Washington, DC Medical Examiners Office |
| Albert et al | protein | 2002 | Sy I ↔ | n/a | n/a | 14 | 14 | WB | 58+-21.5/62+-22.3 | On typical antipsychotics. Patient 6 had taken no neuroleptic drugs for 10 years before death. | 16+-10.5/16+-10.3; NS | C: 12/2/0 S: 10/0/4 | Blind | N/A |
| Nowakowski et al | protein | 2002 | n/a | Sy I ↓ | n/a | 17 | 16 | IHC | 65+-6.3/63+-5.8 | All on antipsychotics | 15+-3.5/17+-3.6; NS | C: 14/2/0 S: 17/0/0 | Not specified | Innsbruck brain collection |
| Talbot et al | protein | 2004 | n/a | Sy I: ↔ | n/a | 17 | 17 | IHC | 44/48 | N/A | 34/24; NS | N/A | Blind | Stanley Foundation Neuropathology Consortium, MD, USA |
| Porton & Wetsel | protein | 2007 | Sy III ↓ | n/a | n/a | 32 | 30 | WB | 42+-8.4/45+-7.6 | N/A | 32+-16/30+-12.2; NS | N/A | Not specified | Stanley Foundation Neuropathology Consortium, MD, USA |

### Supplementary Table 8: Summary of the studies of Rab3A levels in schizophrenia: sample and methodological characteristics and findings

**Legend**:

- **Frontal cortex** to **temporal cortex** fields: graphic depiction of the results for these brain regions. **↔** = no significant change between cases and controls**. ↓** = significant reduction in patients with schizophrenia as compared to controls. ⇣ = absolute levels lower in schizophrenia but did not meet statistical significance on testing; **⇡** absolute levels greater in schizophrenia but did not meet statistical significance on testing
- **N SCZ, N controls**: number of samples per category, SCZ=schizophrenia
- **Technique**: WB = Western blotting, ELISA = enzyme-linked immunosorbent assay, IHC = Immunohistochemistry
- **Mean +-SD of AGE (years) SCZ/CTR**: patients' age +- Standard Deviation/Controls' age +- Standard Deviation
- **Reported SCZ Group Antipsychotic Treatment**: information available in the paper about treatment in the schizophrenia sample
- **Postmortem interval (scz/ctr) hours**: mean in hours for patients/controls (when available); NS = no statistically significant difference
- **Analysis blind to diagnosis?** did the authors report if the biochemical analyses were carried out by a scientist who was blind to sample diagnoses?
- N/A= not available, RT-PCR= Reverse transcription polymerase chain reaction

| **authors** | **year** | **molecule** | **frontal cortex** | **hippocampus** | **Cingulate cortex** | **temporal cortex** | **N, SCZ** | **N, controls** | **technique** | **Age** | **Antipsychotic Treatment in schizophrenia group** | **Mean (sd) postmortem interval (scz/ctr) hours; statistical significance** | **cause of death: number or proportion of suicides** | **analysis blind to diagnosis?** | **source of material** |
| --- | --- | --- | --- | --- | --- | --- | --- | --- | --- | --- | --- | --- | --- | --- | --- |
| Sokolov et al | 2000 | mRNA | n/a | n/a | n/a | **↔** | 14 | 9 | RT-PCR | 77+-3/77+-4 | 4 pts on treatment at time of death | 41+-11/5+-1; p<0.01 | no suicides | Blind | Schizophrenia Brain Bank of the Department of Psychiatry at the Mount Sinai School of Medicine, New York |
| Davidsson et al. | 1999 | protein | **↓** | **↓** | **↓** | **↔** | 5-18 depending on brain region | 6-21 depending on brain region |  | 81+-11.3/75+-12.8 | Yes. Reports mean lifetime dose of neuroleptics | different for different brain areas, overall; NS | controls nil. Patients N/A | Not specified | Department of Psychiatry, Sahlgrenska University Hospital/Molndal, Sweden |
| Blennow | 2000 | protein | **↓** | **↓** | **↓** | **⇣** | 22 depending on brain region | 24 depending on brain region | WB (q) | 79+-10.2/75+-12.3 | Yes. Reports mean lifetime dose of neuroleptics | 42+-18.3/78+-39.8; p=0.001 | all/0/0 | Blind | University of Goteborg |

### Supplementary Table 9: Summary of the studies of synaptotagmin levels in schizophrenia: sample and methodological characteristics and findings

**Legend**:

- **Frontal cortex** to **temporal cortex** fields: graphic depiction of the results for these brain regions. **↔** = no significant change between cases and controls**. ↓** = significant reduction in patients with schizophrenia as compared to controls. ⇣ = absolute levels lower in schizophrenia but did not meet statistical significance on testing; **⇡** absolute levels greater in schizophrenia but did not meet statistical significance on testing
- **N SCZ, N controls**: number of samples per category, SCZ=schizophrenia
- **Technique**: WB = Western blotting, ELISA = enzyme-linked immunosorbent assay, IHC = Immunohistochemistry
- **Mean +-SD of AGE (years) SCZ/CTR**: patients' age +- Standard Deviation/Controls' age +- Standard Deviation
- **Reported SCZ Group Antipsychotic Treatment**: information available in the paper about treatment in the schizophrenia sample
- **Postmortem interval (scz/ctr) hours**: mean in hours for patients/controls (when available); NS = no statistically significant difference
- **Analysis blind to diagnosis?** did the authors report if the biochemical analyses were carried out by a scientist who was blind to sample diagnoses?
- N/A= not available, RT-PCR= Reverse transcription polymerase chain reaction

| **authors** | **year** | **molecule** | **frontal cortex** | **temporal c** | **N, SCZ** | **N, controls** | **technique** | **Age** | **Antipsychotic Treatment in schizophrenia group** | **Mean (sd) postmortem interval (scz/ctr) hours; statistical significance** | **cause of death: number or proportion of suicides** | **analysis blind to diagnosis?** | **source of material** |
| --- | --- | --- | --- | --- | --- | --- | --- | --- | --- | --- | --- | --- | --- |
| Sokolov et al | 2000 | mRNA | n/a | **↔** | 14 | 9 | RT-PCR | 77+-3/77+-4 | 4 pts on treatment at time of death | 41+-11/5+-1; p<0.01 | no suicides | Blind | Schizophrenia Brain Bank of the Department of Psychiatry at the Mount Sinai School of Medicine, New York |
| Gil-Pisa et al | 2012 | protein | **⇣** | n/a | 24 | 24 | WB | 39+-3/39+-2 | 12 treated, 12 antipsychotic free at death | 23+-3/23+-3; NS | C: 0/24 S: 19/24 | Not specified | Basque Institute of Legal Medicine, Bilbao (Spain), and Romand University Center of Legal Medicine – Geneva |
| Ramos-Miguel et al | 2015 | protein | **↔** | n/a | 15 | 13 | WB | Cohort 1: 54 ± 12.1/51 ± 18.8 | Cohort 1: 2 on antipsychotics at death | Cohort 1: 9+-3.4/17+-7.1; p<0.001 | C: 4/9/0 S: 12/3/0 | Blind | Macedonian/New York State Psychiatric Institute Brain Collection |

## Supplementary Figures

### **Supplementary Figure 1:** Flowchart showing the inclusion of studies for meta-analysis.

### **Supplementary figure 2:** Meta-analysis funnel plot of synaptophysin in hippocampus in schizophrenia patients as compared to controls.


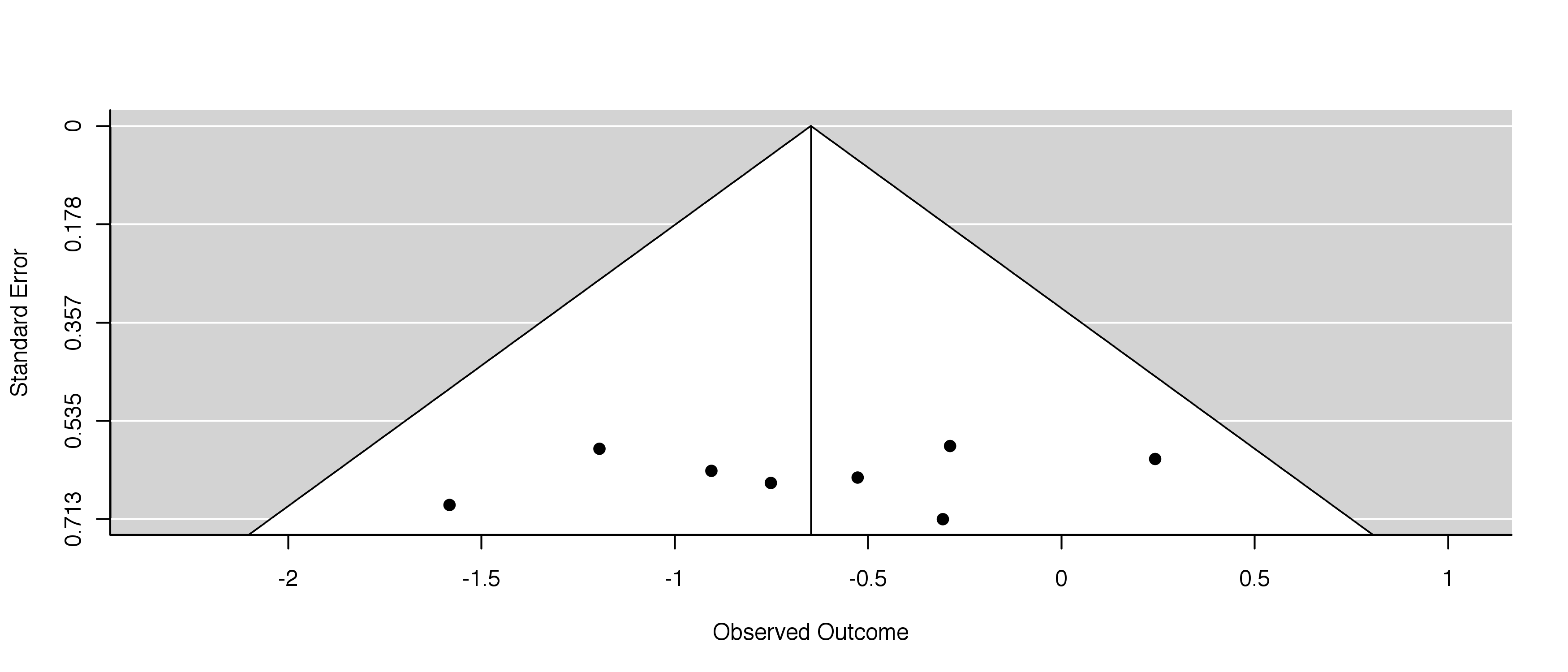


### **Supplementary figure 3:** Meta-analysis funnel plot of synaptophysin in frontal cortex in schizophrenia patients as compared to controls.


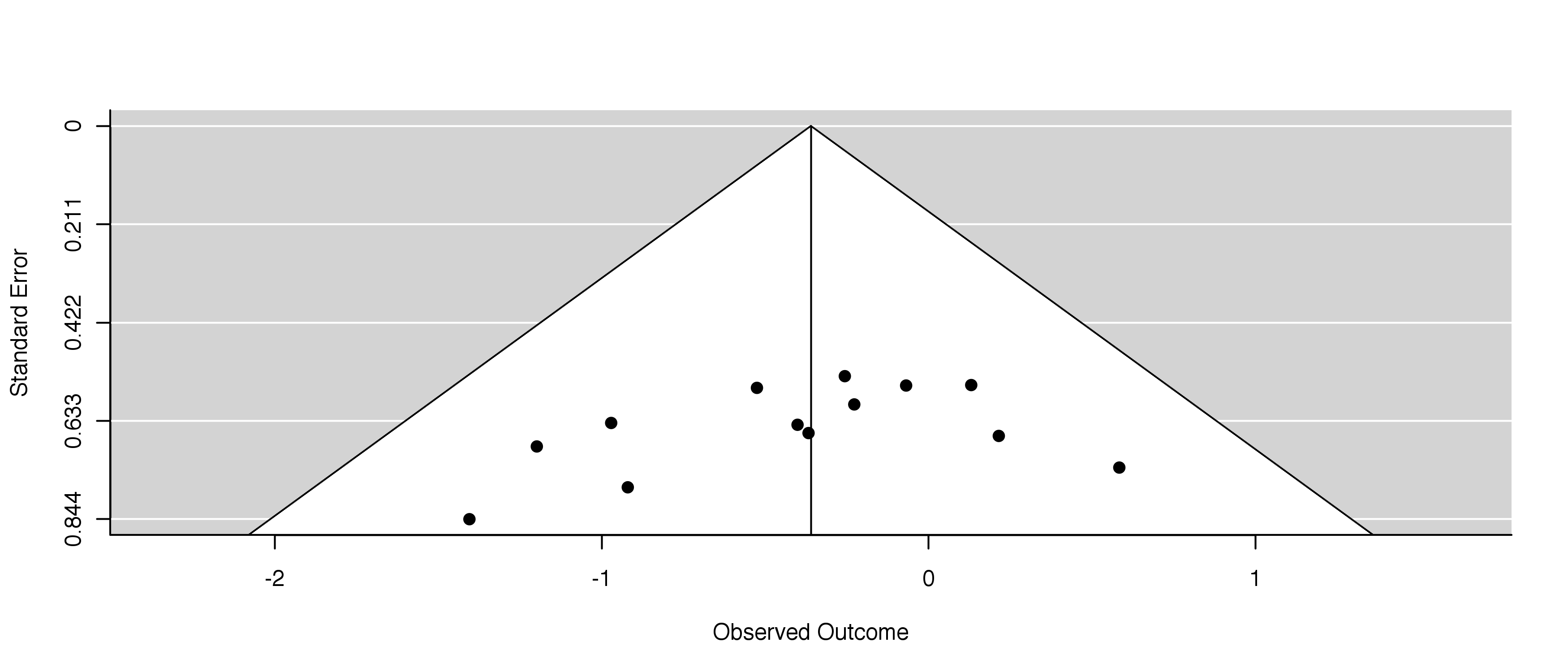


### **Supplementary Figure 4:** Forest plot showing the effect sizes for studies of PSD-95 in frontal cortex in schizophrenia patients as compared to controls. There was no significant reduction in schizophrenia (effect size= -0.34, p= 0.14).


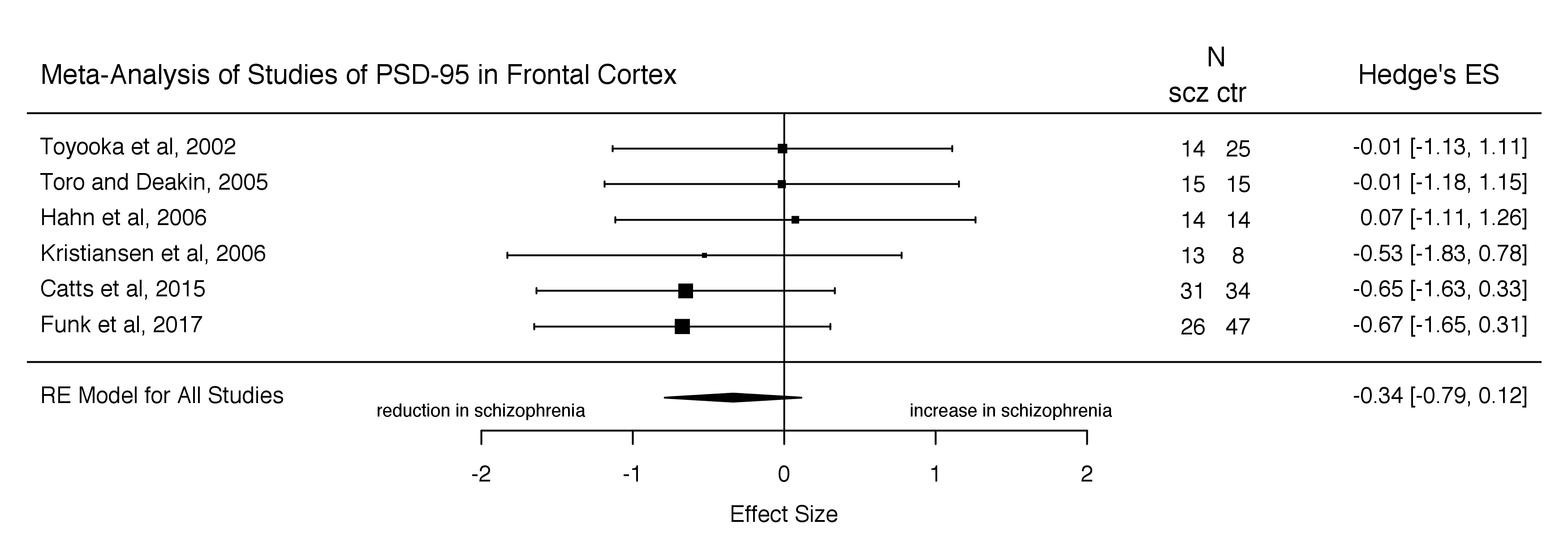


### **Supplementary Figure 5:** Forest plot showing the effect sizes for studies of VAMP in frontal cortex in schizophrenia patients as compared to controls. There was no significant reduction in schizophrenia (effect size= -0.26, p= 0.27).


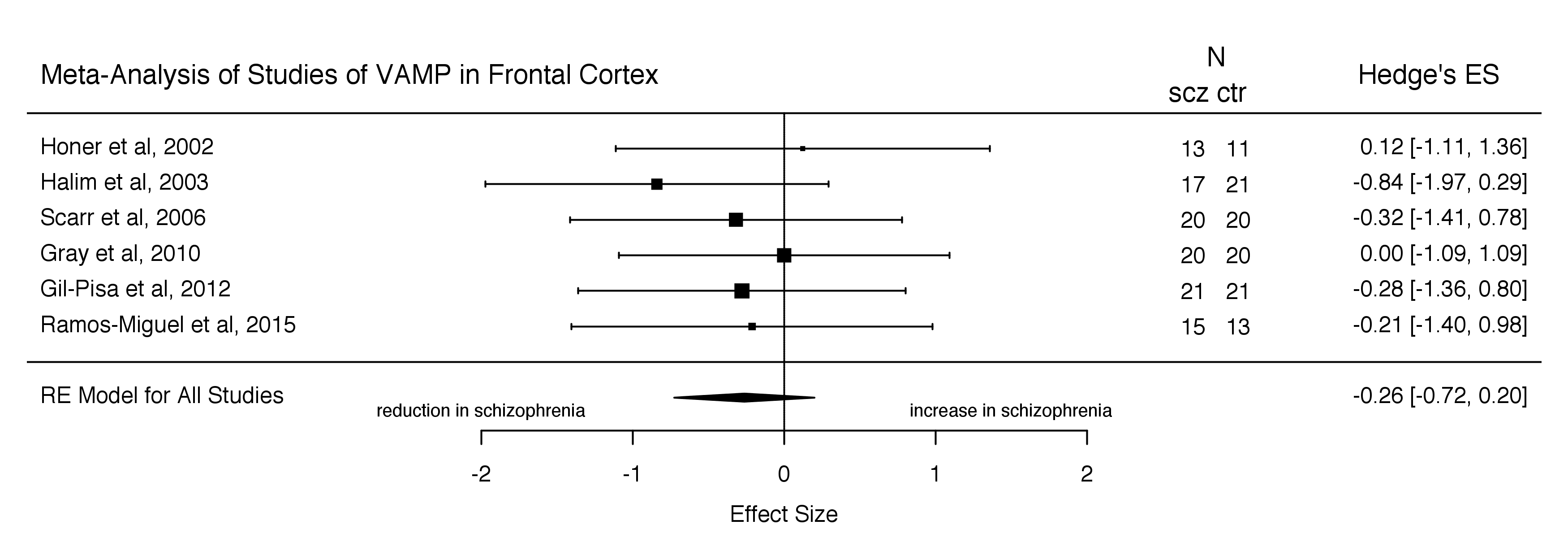


### **Supplementary Figure 6:** Forest plot showing the effect sizes for studies of syntaxin in frontal cortex in schizophrenia patients as compared to controls. There was no significant reduction in schizophrenia (effect size= 0.16, p= 0.52).


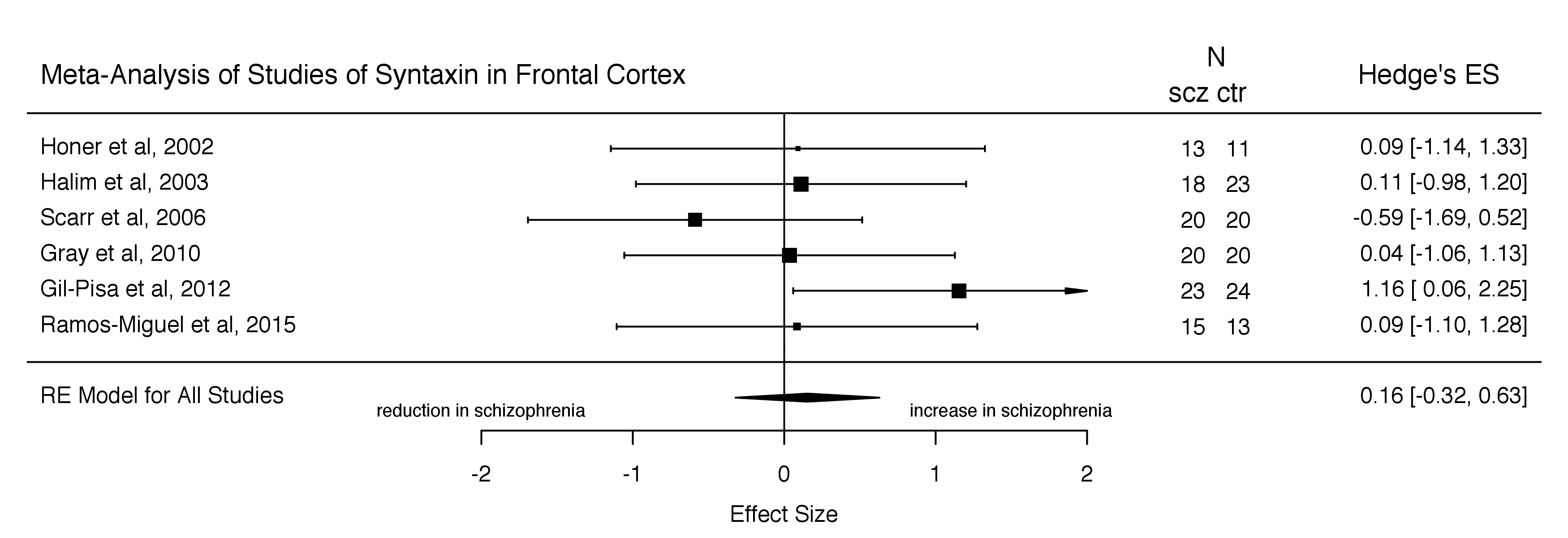


### **Supplementary figure 7:** Meta-analysis funnel plot if synaptophysin in cingulate cortex in schizophrenia patients as compared to controls.


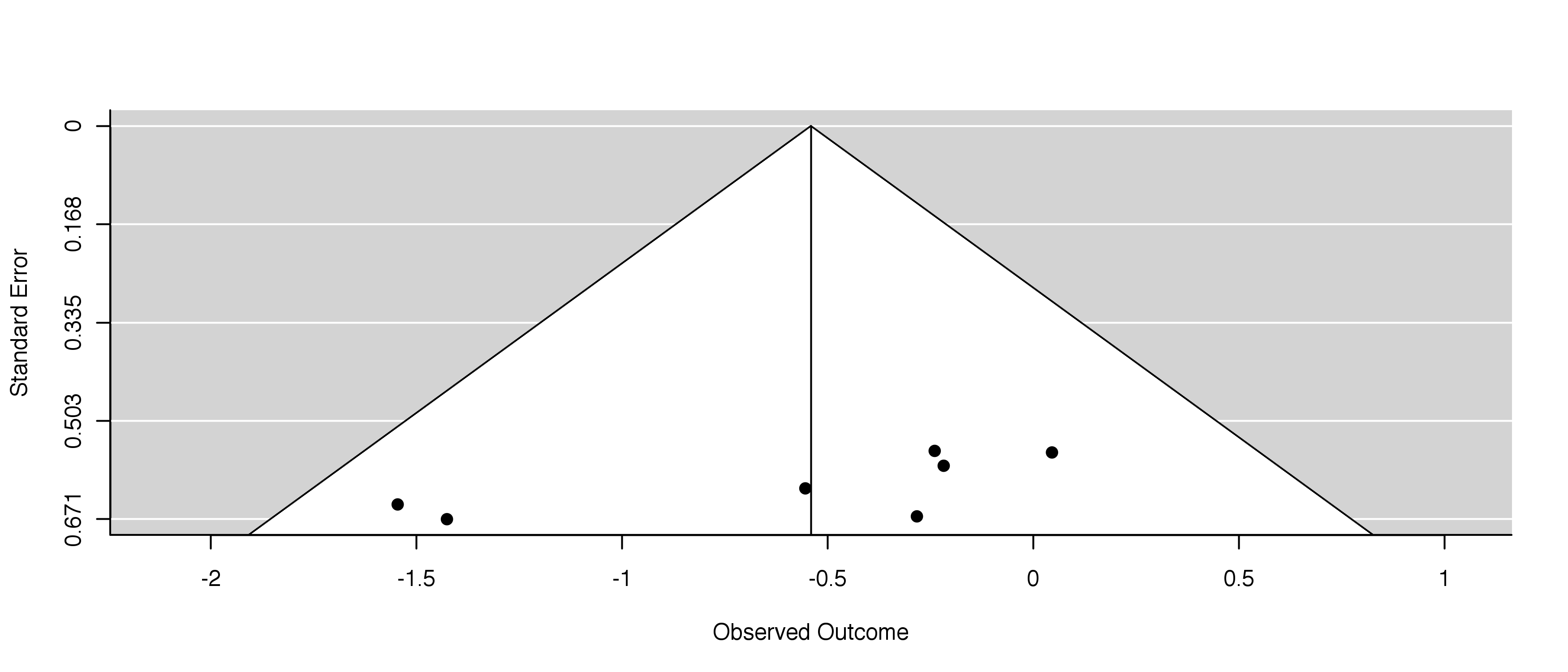


### **Supplementary Figure 8:** Forest plot showing the effect sizes for synaptophysin levels in the temporal cortex in schizophrenia patients as compared to controls. There was no significant reduction in schizophrenia (effect size= -0.31, p= 0.26)


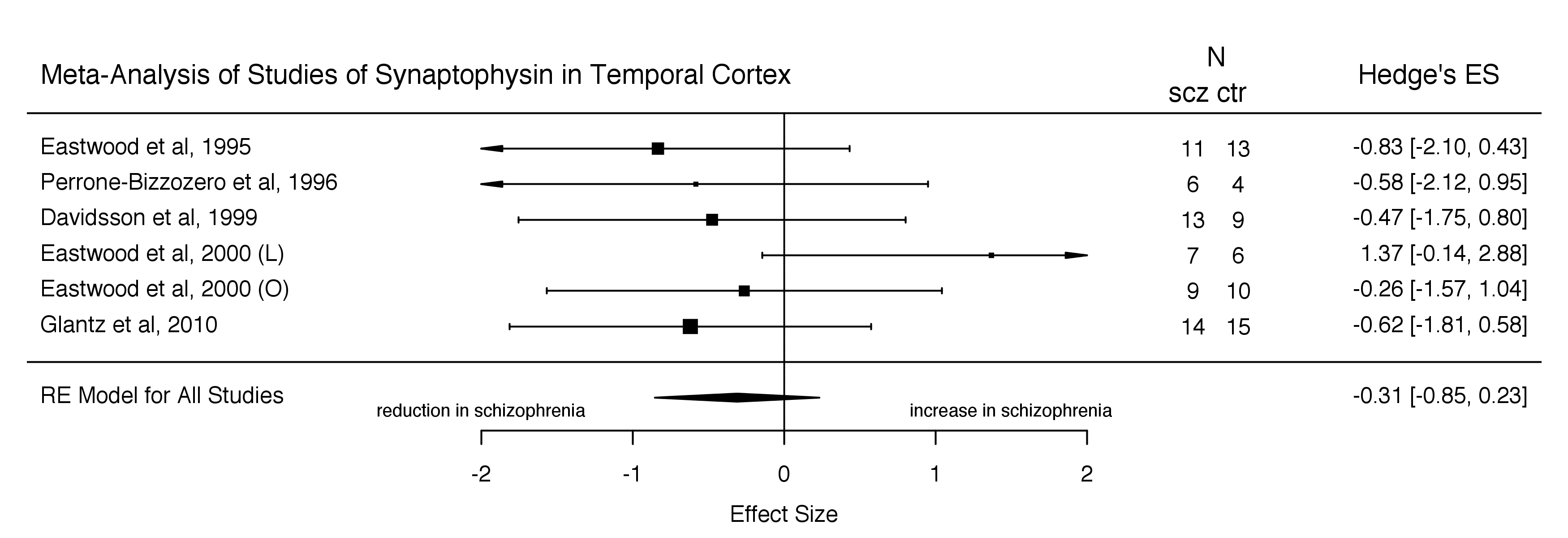


### **Supplementary Figure 9:** Forest plot showing the effect sizes for synaptophysin levels in the occipital cortex in schizophrenia patients as compared to controls. There was no significant reduction in schizophrenia (effect size= -0.16, p= 0.65)


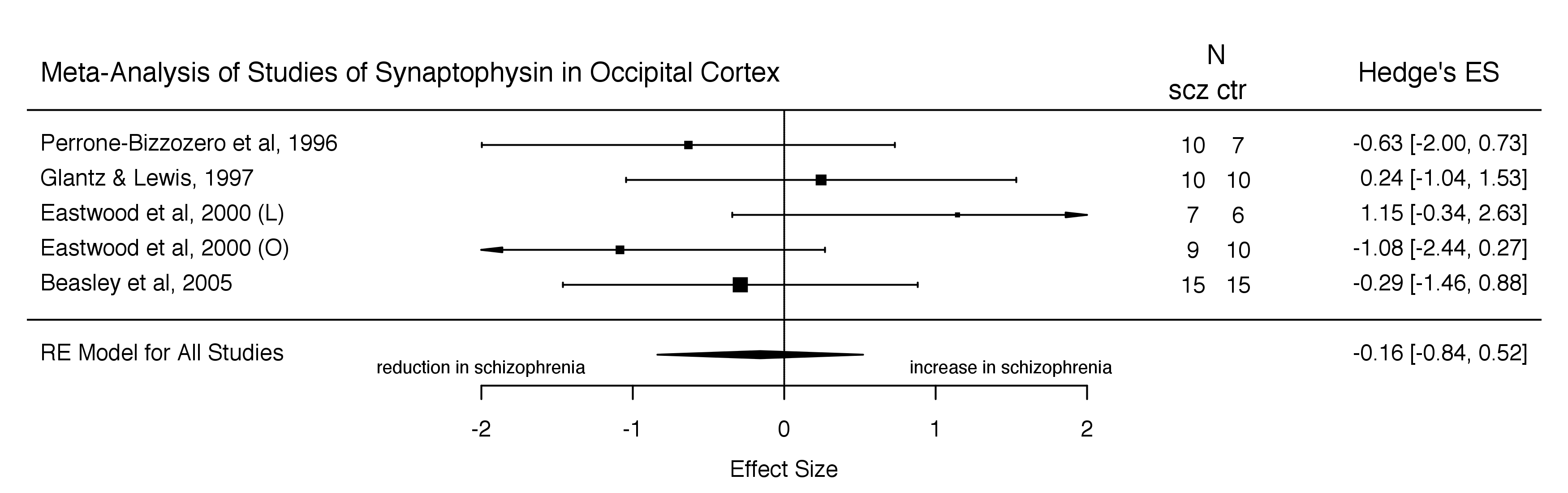


## SUPPLEMENTARY REFERENCES:

1. Castillo MA, Ghose S, Tamminga CA, Ulery-Reynolds PG. Deficits in syntaxin 1 phosphorylation in schizophrenia prefrontal cortex. *Biological psychiatry* 2010; **67**(3)**:** 208-216.

2. Funk AJ, McCullumsmith RE, Haroutunian V, Meador-Woodruff JH. Abnormal activity of the MAPK- and cAMP-associated signaling pathways in frontal cortical areas in postmortem brain in schizophrenia. *Neuropsychopharmacology : official publication of the American College of Neuropsychopharmacology* 2012; **37**(4)**:** 896-905.

3. Talbot K, Louneva N, Cohen JW, Kazi H, Blake DJ, Arnold SE. Synaptic dysbindin-1 reductions in schizophrenia occur in an isoform-specific manner indicating their subsynaptic location. *PloS one* 2011; **6**(3)**:** e16886.

4. WebPlotDigitizer. 2016, Accessed Date Accessed 2016 Accessed.

5. Honer WG, Falkai P, Chen C, Arango V, Mann JJ, Dwork AJ. Synaptic and plasticity-associated proteins in anterior frontal cortex in severe mental illness. *Neuroscience* 1999; **91**(4)**:** 1247-1255.

6. Wan X, Wang W, Liu J, Tong T. Estimating the sample mean and standard deviation from the sample size, median, range and/or interquartile range. *BMC Med Res Methodol* 2014; **14:** 135.

7. Kristiansen LV, Beneyto M, Haroutunian V, Meador-Woodruff JH. Changes in NMDA receptor subunits and interacting PSD proteins in dorsolateral prefrontal and anterior cingulate cortex indicate abnormal regional expression in schizophrenia. *Molecular psychiatry* 2006; **11**(8)**:** 737-747, 705.

8. Hedges LV. Distribution theory for Glass's estimator of effect size and related estimators. *Journal of Educational and Behavioral Statistics* 1981; **6**(2)**:** 107-128.

9. Higgins J, Thompson SG. Quantifying heterogeneity in a meta‐analysis. *Statistics in medicine* 2002; **21**(11)**:** 1539-1558.

10. Marascuilo LA, Serlin RC. *Statistical Methods for the Social and Behavioral Sciences*, vol. 1. W. H. Freeman: New York, 1988, 804pp.

11. Viechtbauer W. Conducting meta-analyses in R with the metafor package. *Journal of Statistical Software* 2010; **36**(3)**:** 1-48.

12. R Development Core Team. R: A language and environment for statistical computing. R Foundation for Statistical Computing: Vienna, Austria, 2008.
